# Resistive force theory and wave dynamics in swimming flagellar apparatus isolated from *C. reinhardtii*†

**DOI:** 10.1039/d0sm01969k

**Published:** 2020-12-09

**Authors:** Samira Goli Pozveh, Albert J. Bae, Azam Gholami

**Affiliations:** Max Planck Institute for Dynamics and Self-Organization Göttingen Germany azam.gholami@ds.mpg.de

## Abstract

Cilia-driven motility and fluid transport are ubiquitous in nature and essential for many biological processes, including swimming of eukaryotic unicellular organisms, mucus transport in airway apparatus or fluid flow in the brain. The-biflagellated micro-swimmer *Chlamydomonas reinhardtii* is a model organism to study the dynamics of flagellar synchronization. Hydrodynamic interactions, intracellular mechanical coupling or cell body rocking is believed to play a crucial role in the synchronization of flagellar beating in green algae. Here, we use freely swimming intact flagellar apparatus isolated from a wall-less strain of *Chlamydomonas* to investigate wave dynamics. Our analysis on phase coordinates shows that when the frequency difference between the flagella is high (10–41% of the mean), neither mechanical coupling *via* basal body nor hydrodynamics interactions are strong enough to synchronize two flagella, indicating that the beating frequency is perhaps controlled internally by the cell. We also examined the validity of resistive force theory for a flagellar apparatus swimming freely in the vicinity of a substrate and found quantitative agreement between the experimental data and simulations with a drag anisotropy of ratio 2. Finally, using a simplified wave form, we investigated the influence of phase and frequency differences, intrinsic curvature and wave amplitude on the swimming trajectory of flagellar apparatus. Our analysis shows that by controlling the phase or frequency differences between two flagella, steering can occur.

## Abstract 2

In basal apparatus isolated from *C. reinhardtii*, if the frequency mismatch is high, mechanical or hydrodynamic coupling cannot synchronize them.

## Graphical abstract

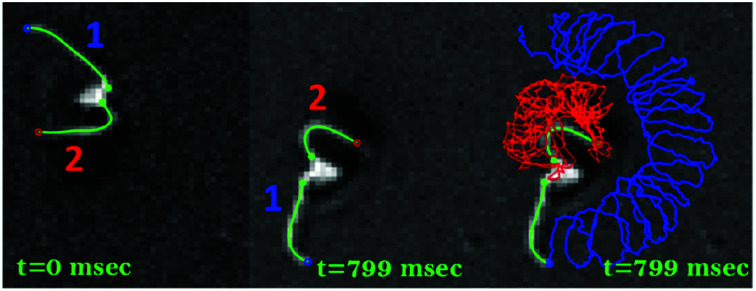

## 1 Introduction

Cilia and flagella are hair-like organelles, which protrude from the surface of many eukaryotic cells and play a fundamental role in signal processing,^1^ sensing,^2,3^ propulsion of micro-organisms^4–6^ and micro-scale fluid transport^7,8^ at a low Reynolds number regime. Cilia and flagella are composed of a microtubule-based structure called the axoneme, and the stresses that lead to the whipping motion are due to dynein molecular motors that exert a force between the microtubule doublets by sliding them with respect to each other, converting chemical energy to mechanical work. Constrains at the basal region and along the contour length of the axoneme convert sliding to bending deformations.^9–11^ The coordinated beating activity of flagella is crucial for efficient swimming of many ciliated cells in an ambient fluid. In response to external stimuli such as light, nutrients, temperature, *etc.*, swimmers transiently change their beating patterns to achieve an efficient directed motion towards the source.

The synchronization mechanism between two or more flagella has been an interesting topic in the past and recent years, attracting the attention of both physicists and biologists. Synchronized beating patterns of two flagella in single-celled microorganisms such as biflagellate green alga *Chlamydomonas reinhardtii* are necessary for a fast directional swimming motion.^12^ The two flagella of *C. reinhardtii* typically beat in synchrony for long time intervals before being interrupted by abrupt large reorientations.^12,13^ It is commonly discussed that interflagellar hydrodynamic interactions between two beating flagella can synchronize their rhythmic patterns.^14–20^ Goldsein *et al.*^12,13^ have analyzed the beats for long time intervals in a series with tens of thousands of beats in micropipette-fixed *Chlamydomonas* cells and observed synchronized states interrupted by phase slips in cells with a small frequency difference of ≃0.1–1%. However, cells with a high frequency mismatch of ≃10–30% beat asynchronously. Using a low-dimensional stochastic model of hydrodynamically coupled oscillators, namely the stochastic Adler equation,^21^ they capture the dynamics of phase slips and the statistics of phase-locked intervals. In this stochastic model, noise amplitude is set by the intrinsic fluctuations of single flagellar beats.^19^ In addition, micropipette experiments by Brumley *et al.*^22^ with somatic cells of *Volvox carteri* confirm that flagella coupled only *via* ambient fluids can achieve full synchronization despite differences in their intrinsic frequencies.

On the other hand, through experiments performed in ref. 23 with *C. reinhardtii*, the hydrodynamic force required for the synchronization of two flagella was measured by applying oscillatory external flows and it was found that the force is more than one order of magnitude larger than hydrodynamic forces experienced under physiological conditions. Furthermore, it has been shown^24,25^ that when the two flagella desynchronize, the rocking of the cell body brings the beating back to synchrony and the contribution of hydrodynamic coupling is negligible to the synchronizing mechanism. However, *C. reinhardtii* cells held fixed with pipettes are also able to synchronize robustly their flagella,^13,26^ thus indicating that synchronization is perhaps due to mechanical coupling *via* internal connecting fibers.^27,28^ In these micropipette-fixed cells, the rate of synchronization measured experimentally is one order of magnitude higher than rates calculated theoretically in the absence of the swimmer movement by only taking into account the direct hydrodynamic interactions.^24^ This support the possibility that either small residual rotational degrees of freedom of the cell body or elastic coupling at the basal ends of two flagella contributes to a rapid synchronization. Remarkably, synchronization in mutants of *C. reinhardtii* missing the filamentous connections is pronouncedly different from wild types.^29^ Other experiments with *C. reinhardtii*^5,12,30,31^ also support the crucial role of mechanical coupling through basal bodies.^23,27^ These fibers have a microtubule-based structure showing periodic striation patterns.^27^ The periodicity (around 80 nm in *C. reinhardtii*) can change in response to chemical stimuli such as calcium ions, indicating the contractility of the fibers.^32^

On the theoretical side, analyses of a three-sphere model in ref. 25, 33 and 34 have demonstrated that for a free swimmer, synchronization can be achieved in the absence of hydrodynamic interactions solely due to local hydrodynamic drag forces which couple oscillatory motion of two flagella *via* movements of the swimmer. Remarkably, in this toy model, in the absence of free translational and rotational motions of the swimmer, synchronization of the flagellar phases becomes relatively weak, and so to achieve synchronization in micro-pipette experiments, elastic coupling at the flagellar base or small residual rotational degrees of freedom are required.

Experiments with the isolated flagellar apparatus from a wall-less mutant of *C. reinhartii* by Hyams and Borisy^35,36^ have shown that both flagella are able to maintain their beating patterns similar to those found in intact cells. Thus, the presence of a cell body or cytoplasm is not necessary to synchronize two flagella and is possibly an intrinsic structural property of basal apparatus. In the flagellar apparatus, the two flagella are connected at an angle forming a V-shape at their basal ends with elastic fibers connecting the two basal bodies^27^ are plausible candidates to mechanically couple the oscillatory motion of two flagella and synchronize them. For convenience in microscopy, Hyams and Borisy mainly studied flagellar apparatus anchored to the debris on the substrate and observed that over 70% show synchronous beating patterns, while the rest beat asynchronously. They also observed transient changes from synchrony to asynchrony.

In this work, we studied synchronization dynamics, using phase contrast microscopy, high-speed imaging rates up to 1000 Hz, and image processing to quantify the beating patterns of flagellar apparatus isolated from wall-less mutant of *C. reinhardtii*. In contrast to experiments by Hyams and Borisy,^35,36^ we examined free-moving flagellar apparatus where these swimmers can easily translate and rotate while the two flagella beat at two different intrinsic frequencies (∼15% of the mean). Unconstrained swimmer movements couple rhythmically beating flagella and this coupling is strongly influenced if the swimmer is constrained in movement by *e.g.* attaching to a substrate or holding in place *via* a micro-pipette. The question we wish to address in this study may be stated as follows: can coupling *via* filamentous fibers, swimmer movement or interflagellar hydrodynamic interactions bring two flagella in synchrony, if there is such a high frequency mismatch? Our phase analysis with freely swimming flagellar apparatus demonstrates that these couplings are too weak to cause frequency entrainment. The phase dynamics of oscillator flagella shows that they effectively act as two isolated pendulums beating at their own intrinsic frequencies. They perturb one another's phases without ever achieving full synchronization. Furthermore, we used our tracked data to check the validity of resistive-force theory (RFT) which neglects long-range hydrodynamic interactions, and found quantitative agreement between RFT simulations and experimental data with a drag anisotropy of ratio 2. Finally, by using a simplified wave form, we performed simulations and analytical approximations to study the swimming motion of flagellar apparatus. This analysis shows that by controlling the frequency or phase differences between two flagella, steering of flagellar apparatus can occur.

## 2 Materials and methods

### 2.1 Isolation of basal apparatus

We used a wall-less mutant of *C. reinhardtii* (strain SAG 83.81) to isolate flagellar apparatus, following the protocol of Hymes and Borisy.^35^ Briefly, we grew 1.5 liters cultures of cells in TAP (Tris-acetate-phosphate) medium at 25 °C in a 14 hours light–10 hours dark illumination cycle to reach cell density of ∼10^6^ cells per mL. Cells are centrifuged at room temperature for 15 min at 200 g and resuspended in 100 mL of HMDEK1 solution at 0 °C (10 mM HEPES, 5 mM MgSO_4_, 1 mM DTT, 0.5 mM EDTA, 25 mM KCL, and pH = 7), spun down again at 800 g for 5 minutes and finally resuspended in 5 mL of HMDEK1. At this step, a small fraction of cells (2–3%) released their flagellar apparatus while most of the cells released single flagella. A subsequent centrifugation at 800 g for 5 minutes sedimented the cell bodies keeping flagella apparatus and single isolated flagella in the supernatant. For reactivation with ATP, we mixed the suspension with an equal volume of HMDEK2 solution (30 mM HEPES, 5 mM MgSO_4_, 1 mM DTT, 0.5 mM EDTA, 25 mM KCL, and pH = 7) supplemented with 2 mM ATP at pH = 7. Note that isolated flagellar apparatus is able to reactivate without de-membranation, since the membrane terminates in the vicinity of the basal region of flagella, leaving open ends for free diffusion of ATP.^35,36^ For observation, 10 μL of solution was infused into 100 μm deep flow chambers, built from cleaned glass and 100 μm thick double-sided tape. The glass surface was blocked using casein solution (from bovine milk, 2 mg mL^−1^) to avoid attachment of basal apparatus to the substrate.

### 2.2 High precision tracking of flagella apparatus

We recorded phase contrast microscopy images of planar swimming basal apparatus at 1000 fps for a duration lasting multiple beating cycles. Phase contrast images are first inverted in intensity and then mean intensity (obtained by averaging over the entire video) is subtracted to increase the signal to noise ratio.^37^ A Gaussian filter is applied to smooth the images. In these processed images, the basal body appears as a bright sphere which complicates the tracking procedure of two flagella. Therefore, we removed the basal body by a thresholding step and performed the tracking for each flagellum separately, using a gradient vector flow (GVF) technique.^38,39^ In this method, for the first frame, we select a region of interest which contains basal apparatus with two flagella (see Fig. S1, ESI†). Then, we initialize the snake by drawing a line polygon along the contour of one flagellum in the first frame. This polygon is interpolated at *N* equally spaced points and used as a starting parameter for the snake. The GVF is calculated using the GVF regularization coefficient *μ* = 0.1 with 20 iterations from the image smoothed by a Gaussian filter. The snake is then deformed according to the GVF where we have adapted the original algorithm by Xu and Prince for open boundary conditions.^38,39^ We repeated this procedure separately for each flagellum which gives us positions of *N* points along the contour length *s* of each flagellum so that *s* = 0 corresponds to the basal end and *s* = *L* is the distal tip. Here *L* is the contour length of flagellum. The position of each flagellum at *s*_*i*_ is denoted by **r**_1,2_(*s*_*i*_,*t*) = (*x*_1,2_(*s*_*i*_,*t*),*y*_1,2_(*s*_*i*_,*t*)). Indices 1 and 2 refer to first and second flagellum of basal apparatus.

### 2.3 Shape analysis

The basal apparatus has two flagella, and we performed the mode analysis separately for each flagellum. We described the shape of each flagellum by its unit tangent vector **t̂**(*s*) and the unit normal vector **n̂**(*s*) at distance *s* along the contour. Instantaneous deformation of flagella is described by curvature *κ*(*s*,*t*), which using Frenet–Serret formulae is given by:^40^1
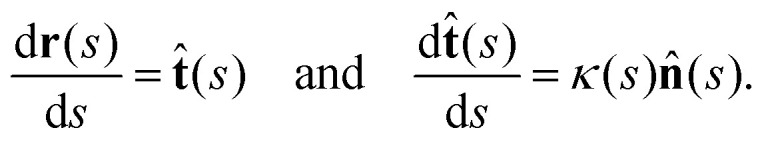


Let us define *θ*(*s*) to be the angle between the tangent vector at distance *s* and the *x*-axis, then *κ*(*s*) = d*θ*(*s*)/d*s*. For shape analysis, we translate and rotate each flagellum such that the basal end is at (0,0) and the orientation of the tangent vector at *s* = 0 is along the *x̂*-axis *i.e. θ*(*s* = 0,*t*) = 0. Following Stephens *et al.*,^40^ we performed principal mode analysis by calculating the covariance matrix of angles *θ*(*s*,*t*) defined as *C*(*s*,*s*′) = 〈(*θ*(*s*,*t*) − 〈*θ*〉)(*θ*(*s*′,*t*) − 〈*θ*〉)〉. The eigenvalues *λ*_*n*_ and the corresponding eigenvectors *V*_*n*_(*s*) of the covariance matrix are given by 
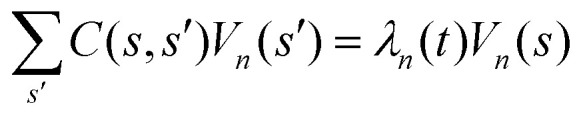
. We show that the superposition of four eigenvectors corresponding to four largest eigenvalues can describe the flagella's shape with high accuracy (see Fig. S2, ESI†):2
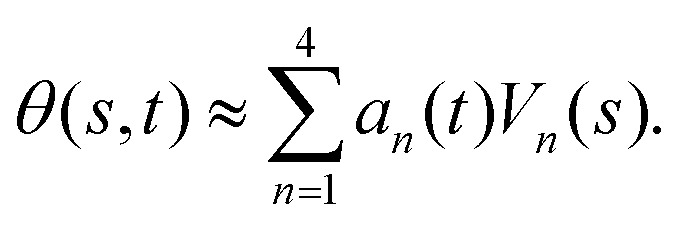
Here the four variables *a*_1_(*t*),…, *a*_4_(*t*) are the amplitudes of motion along different principal components and are given by 
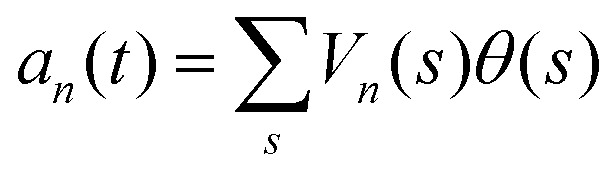
. The fractional variance of flagella's shape captured by *n* eigenvectors is calculated as 
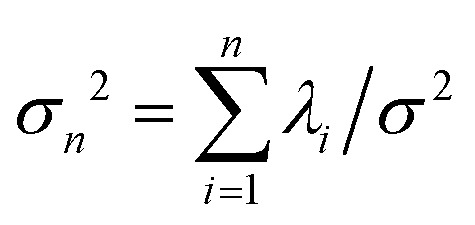
 where 
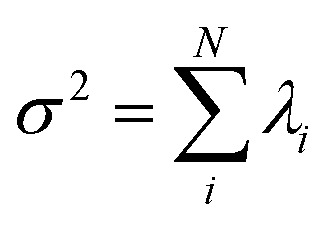
. Here *N* is the total number of eigenvectors. Fig. S2 (ESI†) shows that already two modes capture 96% and four modes capture 99% of the total variance.

### 2.4 Resistive force theory

As basal apparatus (BA) swims in a fluid, it generates flows of Reynolds number of the order of 10^−3^ or less. Thus, in the absence of inertia, the total force **F**^BA^ and torque **T**^BA^ is zero. The total force **F**^BA^ includes friction forces acting on the basal body **F**^BB^ as well as total hydrodynamics forces on both flagella:3
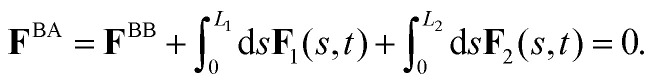
Here *L*_1_ and *L*_2_ are contour lengths of first and second flagella, respectively. Similarly, the total hydrodynamic torque acting on basal apparatus should vanish:4
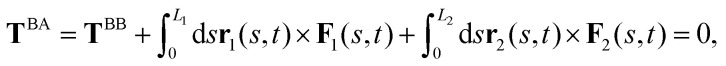
where **T**^BB^ is the torque acting on the basal body. The basal apparatus as a swimmer has two deformable flagella, but at any instant of time it may be considered as a solid body with unknown translational and rotational velocities **U**(*t*) and **Ω**(*t*) yet to be determined. At small Reynolds number, Newton's law becomes an instantaneous balance between external and fluid forces and torques exerted on the swimmer:

5 **F**^ext^ + **F**^BA^ = 0, **T**^ext^ + **T**^BA^ = 0,

where the force **F**^BA^ and torque **T**^BA^ exerted by the fluid on the basal apparatus are given in eqn (3) and (4) and can be separated into propulsive part due to the relative deformation of both flagella in body-fixed frame and drag part:^41^6
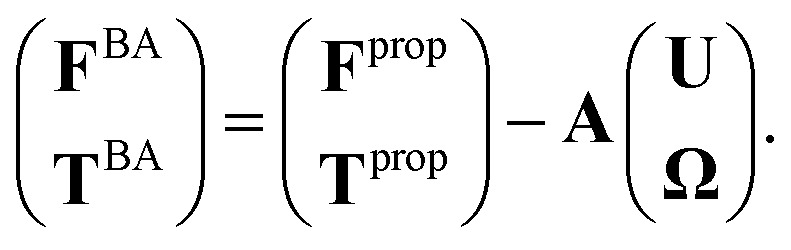
Here *A* is a 6 × 6 drag coefficients matrix which is symmetric and nonsingular (invertible) and it depends only on the geometry of basal apparatus. In our analysis, two flagella and basal body are considered as one long flagellum which is attached to a spherical basal body at its middle. As mentioned above, since in our system, no external forces and torques are acting on basal apparatus, therefore **F**^BA^ and **T**^BA^ must vanish. Furthermore, since basal apparatus swims effectively in 2D, **A** is reduced to a 3 × 3 matrix and eqn (6) can be written as:7
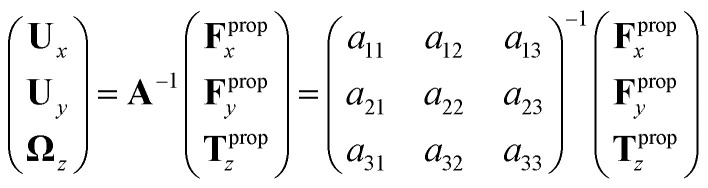


We determine the elements of drag matrix **A** by computing the propulsive force and torque exerted by fluid on the swimmer in the lab frame for a translating non-rotating and for a rotating non-translating basal apparatus.

To determine **F**^prop^_*x*_, **F**^prop^_*y*_ and **T**^prop^_*z*_ which are propulsive forces and torque due to shape deformations of two flagella in body-fixed frame, we first define a reference frame which is fixed with respect to some arbitrary reference point in basal apparatus, namely the basal body. We set the origin of the body-fixed frame at basal body and define the local tangent vector of first flagellum at contour length *s* = 0 as *X̂* direction, the local normal vector *n̂* as *Ŷ* direction, and assume that *ẑ* and *Ẑ* are parallel. We let *Φ*(*t*) = *θ*(*s* = 0,*t*) to be the angle between *x̂* and *X̂* (see Fig. 2A), then in the laboratory frame the velocity of basal body, which was defined as the origin of the body-fixed frame, is given by:8
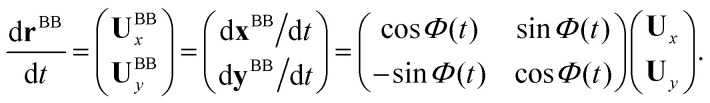
Furthermore, the instantaneous velocity of each flagellum in the lab frame is given by **u** = **U** + *Ω* × **r**(*s*,*t*) + **u**′, where **u**′ is the deformation velocity of flagella in the body-fixed frame, *U* = (**U**_*x*_,**U**_*y*_,0) and *Ω* = (0,0,*Ω*_*z*_) with *Ω*_*z*_ = d*Φ*(*t*)/d*t*.

To calculate **F**^prop^_*x*_, **F**^prop^_*y*_ and **T**^prop^_*z*_ for a given beating pattern of each flagellum in body-fixed frame, we used classical framework of resistive force theory (RFT) which neglects long-range hydrodynamic interactions between different parts of each flagellum as well as inter-flagella interactions. In this theory, each flagellum is divided to small cylindrical segments moving with velocity **u**′(*s*,*t*) in the body-fixed frame and the propulsive force **F**^prop^ is proportional to the local centerline velocity components of each segment in parallel and perpendicular directions:9
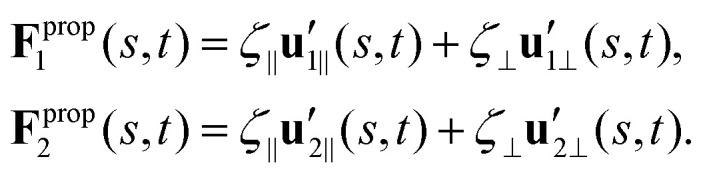
where indices 1 and 2 refer to first and second flagellum in basal apparatus. Here 
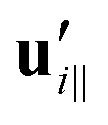
 and 
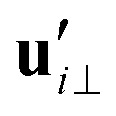
 are projections of the local velocity on the directions parallel and perpendicular to each flagellum, *i.e.*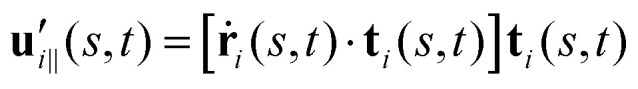 and 
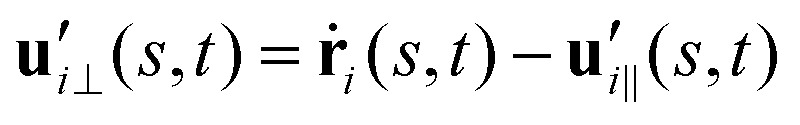
 (*i* = 1, 2) where **t**_1,2_ are the unit tangent vectors along the first and second flagella. The friction coefficients *ζ*_‖_ and *ζ*_⊥_ for a thin filament of contour length *L* ∼ 10 μm and radius *a* ∼ 100 nm swimming in a surrounding fluid with viscosity *μ* = 0.96 pN ms μm^−2^ (water at 22 °C) are given by *ζ*_‖_ = 4π*μ*/(ln(2*L*/*a*) + 0.5) ∼ 2.1 pN ms μm^−2^ and *ζ*_⊥_ = 2*ζ*_‖_. This anisotropy indicates that to obtain the same velocity, one would need to apply a force in the perpendicular direction twice as large as that in the parallel direction.^42^ Furthermore, in the lab frame, the basal ends of both flagella move in synchrony with the basal body *i.e.***u**_1‖_(*s* = 0,*t*) = **u**_2‖_(*s* = 0,*t*) = **U**^BB^_‖_ and **u**_1⊥_(*s* = 0,*t*) = **u**_2⊥_(*s* = 0,*t*) = ***U***^BB^_⊥_.

The effects of basal body connected to the basal ends of both flagella are accounted for by defining **F**^BB^ and **T**^BB^ in eqn (3) and (4) to be the drag force and torque acting on the basal body and are given as:
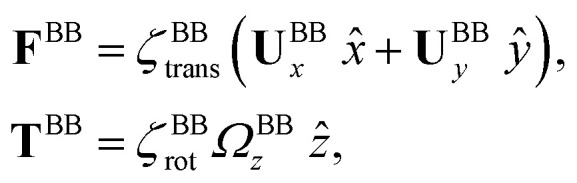
where **U**^BB^ and *Ω*^BB^_*z*_ denote the translational and rotational speed of basal body in the lab frame. In our analysis, we consider the basal body to be a sphere of dimensionless size *b* = 0.1 which is the ratio between its radius (∼1 μm) and contour length of flagella (∼10 μm). We calculate the translational and rotational friction coefficients of basal body as *ζ*^BB^_trans_ = 6π*α*_t_*μb* and *ζ*^BB^_rot_ = 8π*α*_r_*μb*^3^, where factors *α*_t_ = (1 − (9/16)(*b*/*h*) + (1/8)(*b*/*h*)^3^)^−1^ and *α*_r_ = (1 − (1/8)(*b*/*h*)^3^)^−1^ are corrections due to the fact that basal apparatus swims in the vicinity of a substrate.^43^ Here *h* is the distance between the center of the sphere (basal body) and the substrate, non-dimensionalized to the contour length of flagellum. Assuming the case where the basal apparatus swims in the immediate vicinity of the substrate, *i.e. b*/*h* = 1, we obtain *α*_t_ = 16/9 and *α*_r_ = 8/7.

Here is a short summary of steps in our RFT analysis: first, we translate and rotate the basal apparatus such that basal body is at position (0,0) and the local tangent vector of first flagella at *s* = 0 and time *t* is in the direction defined by the tangent vector of first flagella at *t* = 0 and *s* = 0 (*X̂*-axis in Fig. 2A). In this way, we lose the orientation information of the basal apparatus at all the time points except for the initial configuration at time *t* = 0. Note that the isolated flagellar apparatus maintains the V configuration characteristic of the apparatus *in situ*,^27^ therefore the angle of V-shape configuration at the basal ends of two flagella which changes over time is an input from our experimental data used for simulations. Second, we calculate the propulsive forces and torque in the body-frame using RFT and eqn (7) to obtain translational velocities **U**_*x*_ and **U**_*y*_ as well as rotational velocity *Ω*_*z*_ of basal apparatus. Now the rotational matrix can be calculated as:10
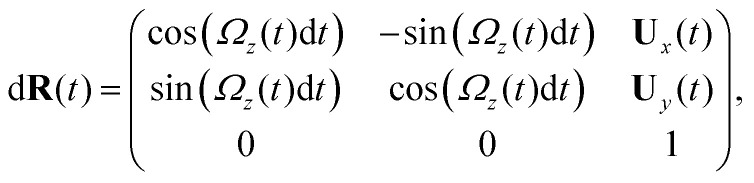
which we use to update the rotation matrix as **R**(*t* + d*t*) = **R**(*t*)d**R**(*t*), considering **R**(*t* = 0) to be the unity matrix. Having the rotation matrix at time *t*, we obtain the configuration of basal apparatus at time *t* from its shape at body-fixed frame by multiplying the rotation matrix as **r**_lab-frame_(*s*,*t*) = **R**(*t*)**r**_body-frame_(*s*,*t*), which can then be compared with experimental data. Note that **r**_body-frame_(*s*,*t*) is an input from experimental data presenting the beating patterns in the body-fixed frame.

## 3 Results

### 3.1 Wave form and beating frequencies

**Fig. 1:**
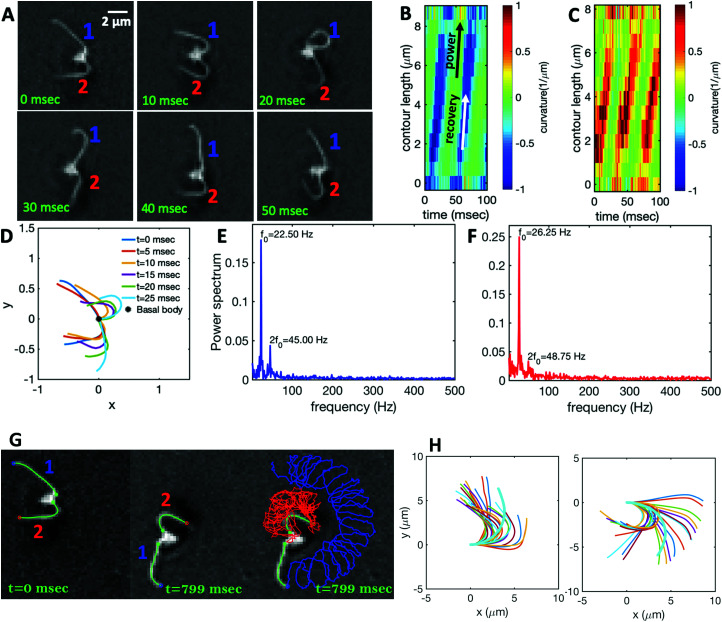
(A) Sample snapshots of swimming isolated flagellar apparatus. (B and C) Curvature waves propagating along the contour length of both flagella showing power and recovery strokes (see also Fig. S4, ESI†). (D) Representative flagellar waveforms, while basal body is translated to be at (0,0). (E and F) Power spectral density of both flagella showing that the first one beats at a frequency of 22.50 Hz, while the second one beats faster at a frequency of 26.25 Hz. (G) Swimming flagellar apparatus shown at two different time points. Both flagella are tracked using the GVF method. Trajectories of distal ends of both flagella as it swims in a time interval of 0 to 799 ms are shown in the last panel. (H) The basal ends of tracked filaments of both flagella are translated to position (0,0) and rotated such that the tangent vector at *s* = 0 is along the *x*-axis. Semi-circular arcs in cyan color with mean curvatures of *κ*_0_ ∼ 0.285 μm^−1^ and 0.268 μm^−1^ show the time-averaged shape of flagellum 1 and 2, respectively. This averaged intrinsic curvature makes the waveform asymmetric (see Video S1, ESI†).

**Fig. 2:**
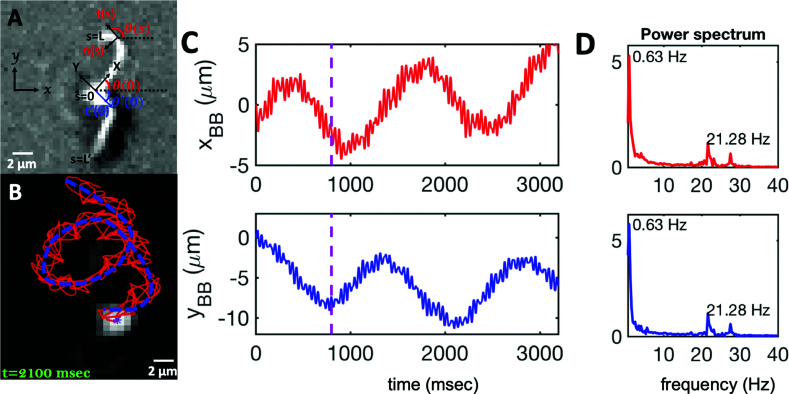
(A) Definition of laboratory axes *x*, *y* and body-fixed axes *X*, *Y*. (B) Wiggling movement of the basal body on a helical trajectory as it swims with its two flagella in the vicinity of a substrate. (C) Displacements of basal body *x*_BB_ and *y*_BB_ relative to the original position at *t* = 0 showing small-scale oscillations with a frequency of 21.28 Hz (beating frequency of flagella) embedded in large-scale oscillations of frequency around 0.63 Hz. The dashed lines in magenta highlight the first 800 frames that are used for tracking in Fig. 1. (D) Power spectrum of *x*_BB_ and *y*_BB_ displays clear peaks at two frequencies of 21.28 and 0.63 Hz (see Video S2, ESI†).

Fig. 1A illustrates the planar swimming motion of an isolated flagellar apparatus immersed in a water-like fluid supplemented with 2 mM ATP. If we define the posterior as the basal end of the apparatus, it swims forward as bending waves propagate from basal regions towards the distal tips (Fig. 1B and C). The flagella beat with a power stroke and a recovery stroke comparable to those observed in intact *Chlamydomonas* cells (see Fig. S3, ESI†). We tracked each flagellum separately using the GVF method (see Fig. 1D and Section 2.2) to characterize the curvature waves and beating frequencies (Fig. 1E and F). Traces of the distal ends of both flagella are shown in Fig. 1G. This method gives us *x* and *y* coordinates of *N* points along each flagellum which is used to calculate the angle between the local tangent vector and *x*-axis, *θ*(*s*,*t*) (see Fig. 2A).

We quantified the curvature waves propagating along the contour length of each flagellum using tangent angle *θ*(*s*,*t*) (Fig. 1B and C). FFT analysis of curvature waves shows dominant peaks at 22.50 Hz and 26.25 Hz for first and second flagellum, respectively (Fig. 1E and F). This is smaller than the typical beating frequencies of flagella in intact wall-less mutants of *Chlamydomonas* cells (see Fig. S3, ESI†). We also observed clear peaks at the second harmonics (45 Hz and 48.75 Hz) which temporally break the mirror symmetry of beating patterns.^44^ Interestingly, the oscillatory pattern of each flagellum exhibits a pronounced asymmetry, corresponding to a constant static curvature.^45,46^ We calculated the time-averaged shape of each flagellum which results in a semi-circular arc with an intrinsic curvature of *κ*_0_ ∼ π/*L* ∼ 0.24 rad μm^−1^. Here *L* ∼ 10 μm is the contour length of the flagellum (see Fig. 1H). This value of *κ*_0_ is comparable to the results reported for axonemes isolated from wild type *C. reinhardti* cells,^46^ as well as our analysis of mean curvature of flagella in intact wall-less *C. reinhardti* cells (see Fig. S3, ESI†).

As the flagellar apparatus swims, the basal body follows a helical path as displayed in Fig. 2B. The basal body trajectory shows a wiggle with a frequency of 21 Hz embedded in large-scale oscillations with much smaller frequency of 0.63 Hz (Fig. 2C and D). Note that a flagellar apparatus with two flagella beating exactly at the same wave amplitude, frequency and phase, swims in a straight path. However, variations in these parameters generate torque causing the basal apparatus to swim in a circular path (see Section 3.3). Even if two flagella beat at the same frequencies, the phase and amplitude of curvature waves are dynamic variables, so the swimmer moving on a curved path does not return precisely to its initial position and follows a helical trajectory.

### 3.2 Mode analysis of flagellar shape and phase dynamics

**Fig. 3:**
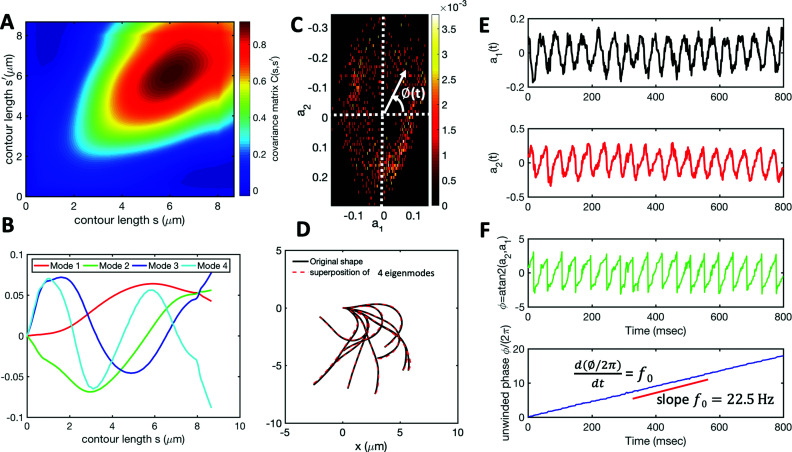
Mode analysis of first flagellum of basal apparatus. (A) The covariance matrix *C*(*s*,*s*′) of fluctuations in angle *θ*(*s*,*t*). (B) Four eigenvectors corresponding to four largest eigenvalues of matrix *C*(*s*,*s*′). (C) Probability density of the first two shape amplitudes *P*(*a*_1_(*t*),*a*_2_(*t*)). The phase angle is defined as *ϕ*(*t*) = atan2(*a*_2_(*t*),*a*_1_(*t*)). (D) Superposition of four eigenmodes presented in part B with coefficients *a*_1_(*t*) to *a*_4_(*t*) can reproduce the shape of flagella with high accuracy (see Fig. S2, ESI†). (E) Time evolution of the first two dominant shape amplitudes *a*_1_(*t*) and *a*_2_(*t*) showing regular oscillations of frequency 22.50 Hz. (F) Dynamics of the phase *ϕ*(*t*) shows a linear growth, indicating steady rotation in the *a*_1_ − *a*_2_ plane presented in part C. We note that d*ϕ*/d*t* = 2π*f*_0_, where *f*_0_ is the beating frequency.

We performed principal mode analysis to describe the time-dependent shape of both flagella in swimming apparatus. This analysis is based on the method introduced by Stephens *et al.*,^40^ which was initially used to characterize wave forms in *C. elegans*. We used *x* and *y* coordinates of each tracked flagellum, obtained using the GVF technique^38,39^ (see Section 2.2), to calculate *θ*(*s*,*t*) which is the angle between the local tangent vector of tracked flagellum center line and *x̂*-axis (see Fig. 2A). Next, we computed the covariance matrix *C*(*s*,*s*′) of fluctuations in angle *θ*(*s*,*t*) for each flagellum separately. Fig. 3A shows the color map of the covariance matrix of the first flagellum which has an effective reduced dimensionality with only a small number of non-zero eigenvalues. Remarkably, only four eigenvectors *V*_*n*_(*s*) (*n* = 1,…, 4) corresponding to the first four largest eigenvalues of *C*(*s*,*s*′) capture the flagellum's shape with high accuracy (see Video S3, ESI†). These four eigenvectors are plotted in Fig. 3B and the first two time-dependent motion amplitudes *a*_1_(*t*) and *a*_2_(*t*) are presented in Fig. 3E. Note that the first two modes, which capture 96% of the total variation (see Fig. S2, ESI†), resemble cosine and sine functions that are in quadrature, and that these first two modes correspond to the lowest frequency behavior in our system, while higher modes correspond to higher harmonics.

The Fourier analysis of oscillating motion amplitudes *a*_1_(*t*) and *a*_2_(*t*) gives clear peaks at 22.50 Hz for first flagellum and 26.25 Hz for the second one. Fig. 1E and F also show the existence of higher harmonics at 45 Hz and 48.75 Hz, respectively. Furthermore, the probability density distribution of *a*_1_(*t*) and *a*_2_(*t*) (Fig. 3C) demonstrates that on average, the principal modes follow a closed trajectory reminiscent of a stable limit cycle, and can be used to define an instantaneous phase as *ϕ*(*t*) = atan2(*a*_2_(*t*),*a*_1_(*t*)). The phase *ϕ* maps variables *a*_1_(*t*) and *a*_2_(*t*) undergoing non-linear oscillations (Fig. 3E) to a new variable which linearly increases over the beating period of each flagellum. By working in phase space, we simply assume that, to leading order, the perturbation due to mechanical coupling *via* the basal body affects only the phase and not the amplitude of the flagella as a non-linear oscillator. Fig. 3F shows the time-dependent phase calculated for first flagellum which is (on average) a monotonically increasing function of time and is equivalent to a uniform rotation in the phase space defined in the *a*_1_ − *a*_2_ plane (Fig. 3C). The time derivative of phase *ϕ*(*t*) is a measure of the oscillation frequency *f*_0_.

**Fig. 4:**
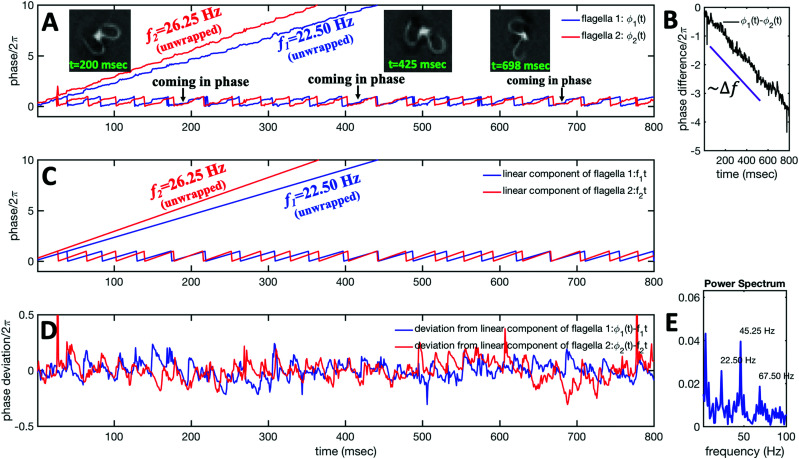
Phase dynamics of two mechanically coupled flagella in swimming basal apparatus. (A) For each flagellum, we extract a time-dependent phase *ϕ*(*t*) from motion amplitudes *a*_1_(*t*) and *a*_2_(*t*) defined as *ϕ*(*t*) = atan2(*a*_1_(*t*),*a*_2_(*t*)). Unwrapped phase for each flagellum shows fluctuations around a linear trend. (B) For a frequency mismatch of 3.75 Hz (15% of the mean), the two flagella display phase differences that vary monotonously with time. (C) Linear phase component of each flagellum that scales with frequency. (D) Deviations from linear components where the unwrapped phase of two flagella in part A is subtracted from the corresponding unwrapped linear components presented in part C. (E) Power spectrum of non-linear fluctuations of the first flagellum showing clear peaks at beating frequency of 22.50 Hz and at higher harmonics.

The phase dynamics of each flagellum is composed of non-linear fluctuations around a linear deterministic time trend (Fig. 4A). If the two flagella were independent non-interacting oscillators, we would expect the phase difference to grow linearly at a rate proportional to the frequency mismatch Δ*f* (Fig. 4B), and besides some minor fluctuations, this is what we see – *i.e.* we find no evidence of phase locking and synchronization. Fig. 4D illustrates the deviation of the phase from the linear component obtained by subtracting the unwrapped phase of each flagellum plotted in Fig. 4A, from the corresponding linear component presented in Fig. 4C. The power spectrum of fluctuations of first flagellum gives clear peaks at 22.50 Hz and higher harmonics, as shown in Fig. 4E, similarly for the second flagellum.

Obviously, mechanical coupling fails to entrain two flagella and on average, they behave as two isolated oscillators with phase evolving at constant rates *ω*_1_ = 2π*f*_1_ and *ω*_2_ = 2π*f*_2_ in time. As two flagella beat with 3.75 Hz frequency difference, over time they find similar phase values (black arrows in Fig. 4A), corresponding to the time points that two flagella for a short time beat in synchrony. The frequency difference quickly drives the system out of the synchronous state to the asynchronous phase, which is the dominant state during the swimming period of flagellar apparatus (see Video S1 and Fig. S5 for a similar analysis of another exemplary basal apparatus, ESI†).

A generic description of synchronization in pairs of coupled oscillators in the presence of noise is provided by the stochastic Adler equation:^13,19,21^11
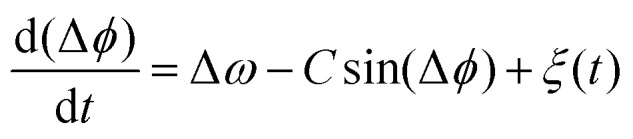
where we can think of d(Δ*ϕ*)/d*t* as the instantaneous frequency difference. Here Δ*ω* = 2π(*f*_2_ − *f*_1_) is the frequency mismatch, *C* is a measure of the coupling strength, and *ξ* = *ξ*_1_ − *ξ*_2_ is a Gaussian white noise which satisfies 〈*ξ*(*t*)〉 = 0 and 〈*ξ*(*t*)*ξ*(*t*′)〉 = 2*T*_eff_*δ*(*t* − *t*′). Indexes 1 and 2 refer to the first and second oscillator and *T*_eff_ indicates an effective temperature.^13^Eqn (11) is commonly used to describe over-damped motion of a massless particle in a periodic potential given by *V*(Δ*ϕ*) = −(Δ*ω*)Δ*ϕ* − *C* cos(Δ*ϕ*). The intrinsic frequency difference Δ*ω* corresponds to a global tilt in this potential. If the frequency mismatch between two oscillators becomes larger than the coupling strength (Δ*ω* > |*C*|), then Adler equation does not have a steady state solution (see Fig. S5F, ESI†). Thus, synchronization cannot occur corresponding to the case of a phase drift where the phase difference depends linearly on time with a slope given by the frequency mismatch Δ*ω*.^12,13,19^ Fluctuations around this linear trend, as shown in Fig. 4B, directly gives a measure of the effective temperature *T*_eff_. In this case, the coupling strength *C*, which includes the effect of hydrodynamic interactions, mechanical coupling and swimmer movement, is much smaller than Δ*ω* and cannot be estimated.^12^ For our experiment presented in Fig. 1 with Δ*ω* = 2πΔ*f* = 23.56 Hz and mean frequency of 〈*ω*〉 = 2π〈*f*〉 = 153.12 Hz, an upper bound of 
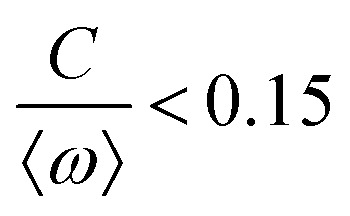
 for the coupling strength can be estimated.

### 3.3 Quantitative agreement with resistive force theory

**Fig. 5:**
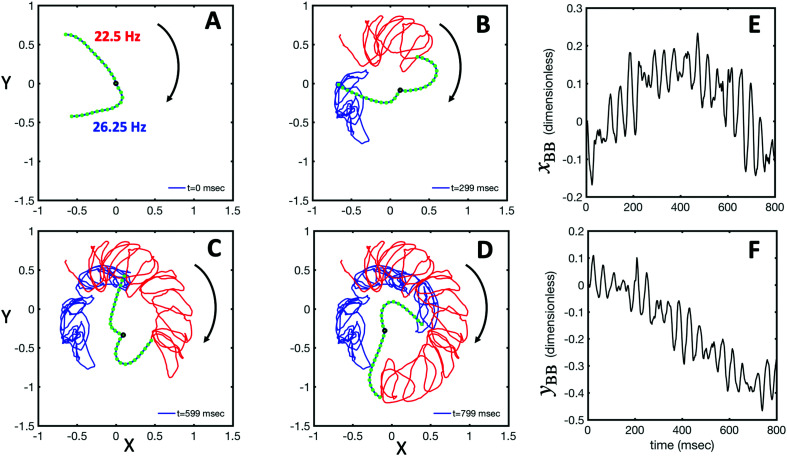
RFT simulations using experimental beating patterns. (A) Initial configuration of flagellar apparatus at *t* = 0 extracted from experimental data (compare with Fig. 1G at *t* = 0). (B–D) Swimming trajectory of flagellar apparatus obtained by RFT simulations. (E and F) Dimensionless positions *x*_BB_ and *y*_BB_ of the basal body obtained from RFT, display oscillations reflecting the beating frequency of flagella. Lengths are non-dimensionalized to the contour length of flagella (see Video S4, ESI†).

**Fig. 6:**
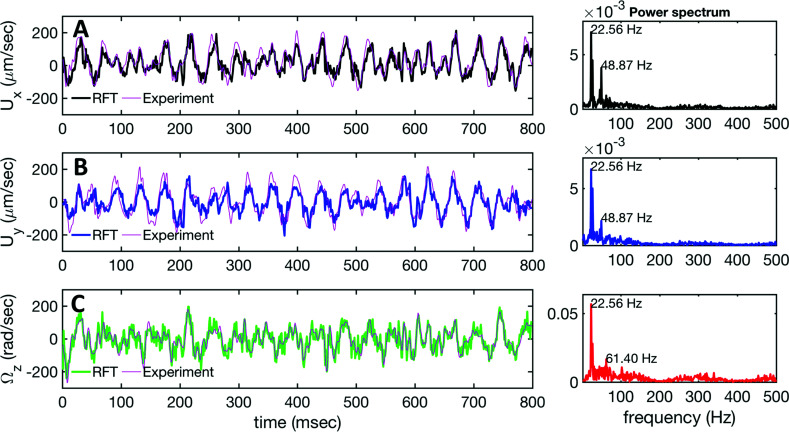
Comparison between RFT analysis and experiments with *ζ*_⊥_/*ζ*_‖_ = 2. (A–C) Instantaneous translational and rotational velocities of basal apparatus **U**_*x*_, **U**_*y*_ and *Ω*_*z*_(*t*) in body-fixed frame, obtained from RFT simulations using experimental beating patterns. Direct experimental results are shown in magenta. Power spectra of translational and angular velocities show clear peaks around 22.50 Hz, which is the beating frequency of the first flagellum.

We used the experimental beating patterns of the flagellar apparatus to examine the validity of resistive force theory in our *in vitro* model system. At each time point *t*, the configuration of flagellar apparatus in the lab frame was calculated from instantaneous translational and rotational velocities of flagellar apparatus in the body-fixed frame using the rotation matrix presented in eqn (10). The control parameter was the drag anisotropy *ζ*_⊥_/*ζ*_‖_ which is the ratio between drag coefficients in perpendicular and tangential directions. Given experimental wave forms, we compared RFT simulations with instantaneous translational and rotational velocities of flagellar apparatus in the body-fixed frame (see Fig. 5 and 6). We found a quantitative agreement for drag anisotropy of *ζ*_⊥_/*ζ*_‖_ = 2. Note that in the body-fixed frame, the motion of basal body is described by time-dependent translational velocities **U**_*x*_, **U**_*y*_ and rotational velocity *Ω*_*z*_ which is a measure of rotation of the basal apparatus. In the lab frame, the translational velocities of the basal body are given by 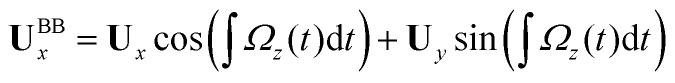 and 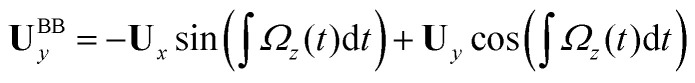. Fig. 6 shows *U*_*x*_, *U*_*y*_ and *Ω*_*z*_ obtained from RFT analysis and a comparison with direct experimental measurements in the body-fixed frame, computed by first differentiating experimental *x*_BB_, *y*_BB_ and *θ*(*s* = 0) with respect to time and then transforming to the swimmer-fixed frame. Remarkably, the corresponding power spectra of *U*_*x*_, *U*_*y*_ and *Ω*_*z*_ show dominant peaks at 22.50 Hz indicating that the beating frequency of the first flagellum is reflected in translational and rotational velocities of the basal body.

#### Simulations with simplified wave forms

To investigate the effect of wave amplitude, frequency and phase difference of two flagella on the swimming trajectory of the basal apparatus, we performed simulations with the simplified form of curvature waves which is a superposition of a dynamic and a static mode:^46^12
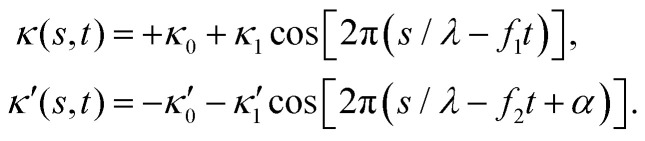
Here *λ* is the wavelength of curvature waves typically on the order of the flagellar length *L*, 2π*α* is the phase difference between two flagellar beats, and constant intrinsic curvature 
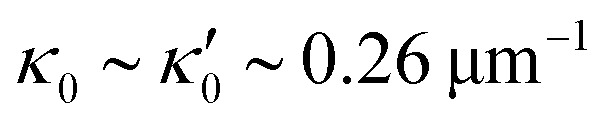
 is determined from experimental data by characterizing the time-averaged shape of each flagellum, as illustrated in Fig. 1H. Furthermore, *κ*_1_ and 
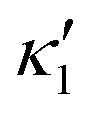
 are the amplitudes of the dynamic modes which are estimated from experimental wave patterns to be around 0.45 μm^−1^. The negative signs in terms *f*_1_*t* and *f*_2_*t* generate curvature waves that propagate from basal regions (*s* = 0) towards the distal tips (*s* = *L*), as observed experimentally. Two flagella are positioned exactly symmetrically with respect to the axis of symmetry of the basal apparatus and in our simulations, we assume the V-junction angle to be fixed at 180°. To perform simulations, we compute the drag force density felt by each flagellum in the framework of resistive-force theory (see eqn (9)). The basal body is assumed to have a dimensionless radius of 0.1.

In general, the mean rotational velocity 〈*Ω*_*z*_〉 of flagellar apparatus depends on the amplitudes of static and dynamic modes as well as the frequency and phase difference between the two flagella. For a single flagellum beating at frequency *f*_0_, in the small-curvature approximation assuming *κ*_0_*L*/(2π) and *κ*_1_*L*/(2π) to be small, 〈*Ω*_*z*_〉 depends linearly on *κ*_0_ but is proportional to the square of *κ*_1_ (see Appendix I, ESI† and ref. 44 and 47):13
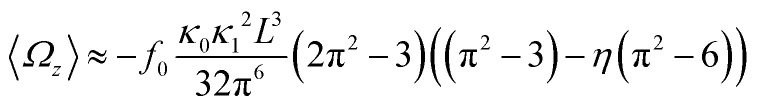
We emphasize that 〈*Ω*_*z*_〉 is non-zero even if the drag anisotropy is zero (*ζ*_‖_ = *ζ*_⊥_). We also note that the mean rotational velocity 〈*Ω*_*z*_〉 scales as *κ*_1_^2^ and therefore is one order of magnitude smaller than instantaneous rotational speed *Ω*_*z*_(*t*) which scales as *κ*_1_.^47–49^

In the case of flagellar apparatus with two flagella, ignoring the hydrodynamic drag force of the basal body for simplicity, rotational velocity has contribution from both flagella (see Appendix I, ESI†):14
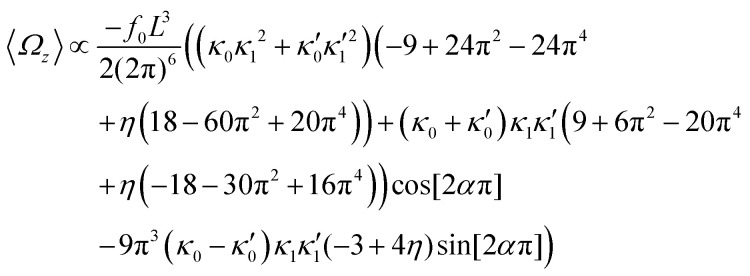
where *η* = *ζ*_‖_/*ζ*_⊥_ and we have assumed the same beating frequency *f*_0_ for both flagella. In the limit of 
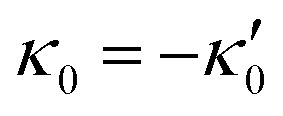
 and 
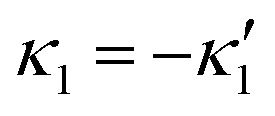
, where the two flagella waveforms are mirror images having same parameters with only a phase difference between them, eqn (14) simplifies to:15
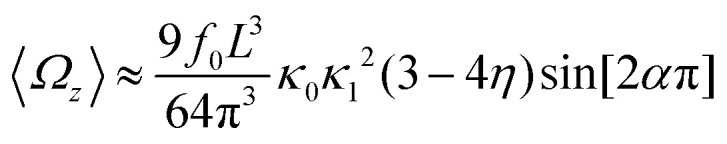
and in the limit of *α* = 0, eqn (14) reduces to:16
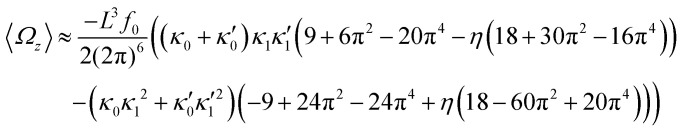


**Fig. 7:**
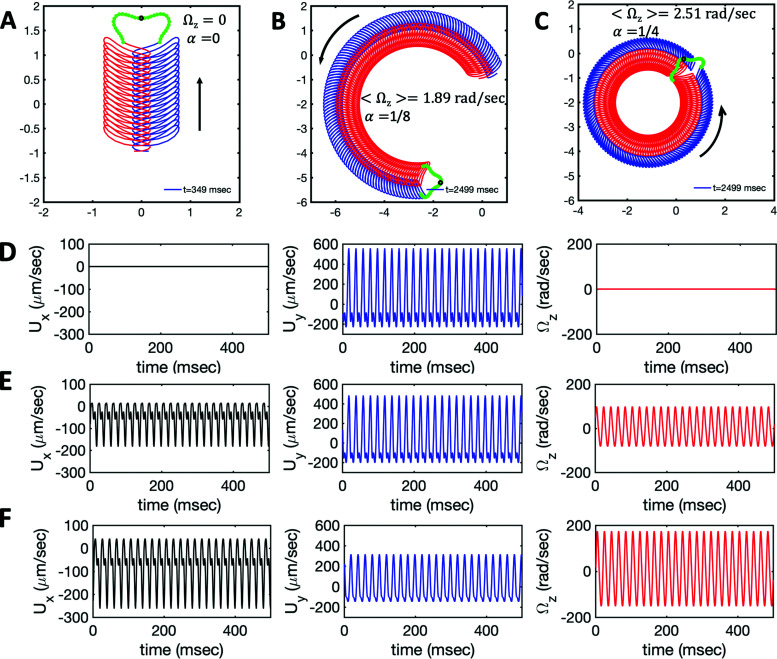
Simulations in the framework of RFT with a simplified wave pattern formed of superposition of a static and a dynamics mode. All parameters are the same for both flagella except the phase shift 2π*α*. (A) Unsteady straight swimming for a flagellar apparatus with *α* = 0. The only non-zero component of velocity is *U*_*y*_ which oscillates over time and there is no rotation. (B) Circular swimming path for swimmer with *α* = 1/8 and (C) *α* = 1/4. The mean rotational velocity is higher for a larger phase shift. (D–F) Instantaneous translational and rotational velocities in body-fixed frame corresponding to parts A, B and C, respectively. Note that both *U*_*x*_, *U*_*y*_ and *Ω*_*z*_ oscillate at a flagellar beat frequency of 50 Hz. Other parameters are: 
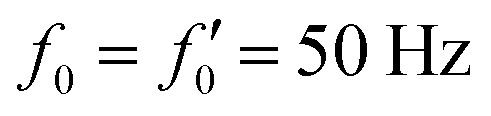
, 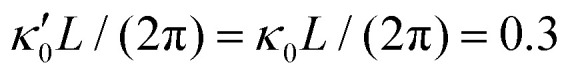, and 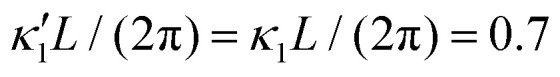. Note that the parameters with prime are for flagella with blue trajectory (see Videos 5–7, ESI†).

**Fig. 8:**
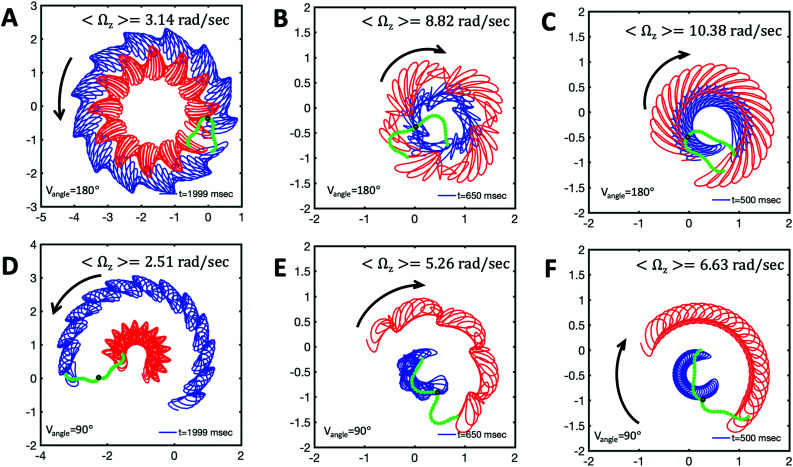
Simulations with simplified wave patterns to study the influence of variations in frequency and amplitude of the dynamic mode *κ*_1_. (A) Flagella with blue trace beats faster compared to the flagellum with red trace (56 Hz *versus* 50 Hz) and therefore it sets the sign of 〈*Ω*_*z*_〉. (B) To switch the direction of rotation, the slower beating flagellum should beat with larger wave amplitude *κ*_1_. (C) Keeping the frequencies and intrinsic curvature similar, flagellum with larger *κ*_1_ (red trace) sets the direction of swimming. Parameters are 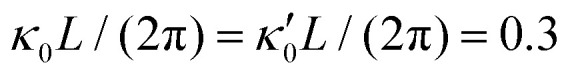, V-junction angle = 180°, *α* = 0 for all simulations, and (A) *f*_0_ = 50 Hz, 
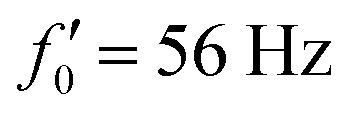
, 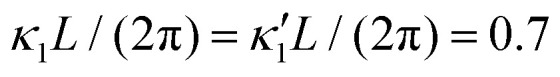, (B) *f*_0_ = 50 Hz, 
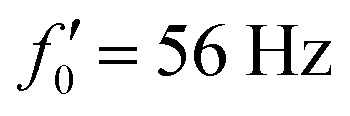
, *κ*_1_*L*/(2π) = 0.7, 
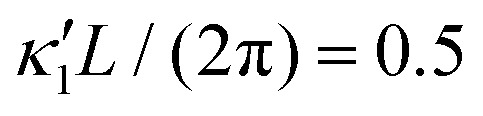
, (C) *f*_0_ = 50 Hz, 
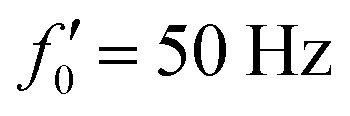
, *κ*_1_*L*/(2π) = 0.7, 
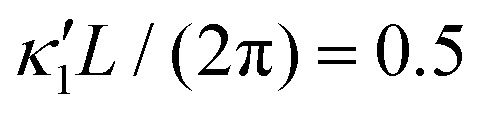
. (D–F) Simulations in parts A–C are repeated with a V-junction angle of 90°. Parameters with prime are for flagella with blue trajectory (see Videos S8–S13, ESI†).

We comment on some properties of eqn (14)–(16): flagellar apparatus with two flagella beating exactly at the same frequency and phase, with mirror-symmetric waveforms (
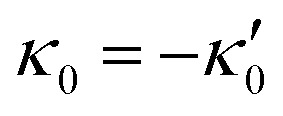
 and 
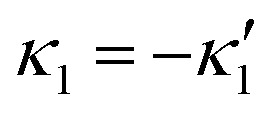
), will swim in a straight path with its translational velocity *U*_*y*_ oscillating at frequency of flagellar beat (Fig. 7A and D). However, only a phase difference between two flagella is enough to change the swimming trajectory to a circular path (Fig. 7B, C, E and F). It is noteworthy that the mean rotational velocity 〈*Ω*_*z*_〉 scales with sin(2π*α*) and thereby, by increasing the phase difference in the range 0 ≤ 2π*α* ≤ π/2, the swimmer rotates on average faster as illustrated in Fig. 7C. At a phase difference between π/2 ≤ 2π*α* ≤ π, 〈*Ω*_*z*_〉 starts to decrease and vanishes at π (*α* = 1/2) (see eqn (15)). Furthermore, a frequency difference between two flagella also generates a circular swimming path. This is shown in Fig. 8A, where two flagella beat at 50 Hz (red trajectory) and 56 Hz (blue trajectory), while all the other parameters are kept the same. Since in our analytical approximation, to calculate the time-averaged *Ω*_*z*_, we consider only one single frequency *f*_0_ (see eqn (S14), ESI†); the influence of having two different beating frequencies is not reflected in our expression in eqn (14). Another point to mention is that the amplitude of dynamic mode enters as *κ*_1_^2^ and 
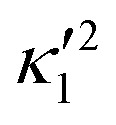
 in the eqn (16) and by introducing a small difference in values of *κ*_1_ and 
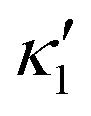
, we can switch the swimming direction. Fig. 8B shows an example of a flagellar apparatus where the flagellum with red trace has a smaller frequency but larger amplitude of the dynamics mode *κ*_1_, while the other flagellum with blue trace has larger frequency but smaller 
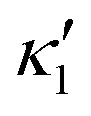
. Thus, the flagellum with larger *κ*_1_ wins and sets the sign of 〈*Ω*_*z*_〉. Remarkably, if the beating frequency, amplitude of dynamic mode and phase are equal for both flagella (
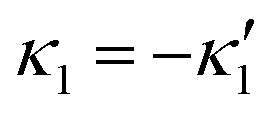
 and *α* = 0), then *Ω*_*z*_ is proportional to 
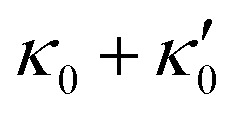
:17
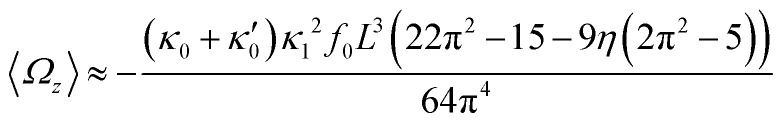
indicating that the flagellum with larger intrinsic curvature sets the swimming direction on the circular path. Furthermore, eqn (14) and (17) show that for an isotropic drag coefficient *ζ*_‖_ = *ζ*_⊥_ (*η* = 1), the rotational velocity is non-zero. Finally, in our simulations, we have assumed that the grafting direction of two flagella at the basal ends, which are positioned exactly symmetrically with respect to the axis of symmetry of basal apparatus, is fixed over time. This angle also affects the mean rotational velocity. All simulations in Fig. 7 and 8A–C (Videos S7–S10, ESI†) are performed with a V-angle of 180°, but we also performed simulations with a V-angle of 90° which is closer to the reported values in ref. 36, 50, and observed a reduction in the mean rotational velocity (Fig. 8D–F and Videos S11–S13, ESI†).

### 3.4 Discussion

In this paper, we have used high-speed imaging, quantitative image processing, and mode analysis to study the wave dynamics of flagellar apparatus isolated from wall-less strain of *C. reinhardtii*. For isolation of this unique *in vitro* system, we followed the protocol established by Hyams and Borisy,^35,36^ which has a very low yield; only 2–3% of the cells release their flagellar apparatus. In contrast to the results reported in ref. 35 and 36, all of the isolated apparatus in our experiments (*N* = 10) had an intrinsic frequency mismatch of Δ*f* = 3.73 ± 1.84 Hz (∼10–41% of the mean) and therefore, it was not possible with our data to investigate the synchronization dynamics in swimmers with no or very small frequency differences. Table S1 in the ESI† presents a summary of frequencies of these 10 basal apparatus. In contrast to previous studies^12,13,26,29^ investigating the synchronization dynamics in micropipette-fixed cells, we focused on freely swimming basal apparatus with an intrinsic interflagellar frequency difference of Δ*f* ∼ 3.75 Hz (∼15% of the mean). In the presence of such a high frequency mismatch, neither rotational motion of the swimmer caused by asynchronous beating of two flagella nor coupling between two flagella either *via* elastic fibers connecting the basal bodies or interflagellar hydrodynamic interactions are strong enough to overcome the high frequency mismatch and the effect of noise (which acts against synchronization) to entrain two flagella. This is in agreement with experimental observations of asynchronous flagellar beats in intact *C. reinhardtii* cells with a high interflagellar frequency difference (10–30%),^12,31,51^ supporting the hypothesis that intrinsic frequencies of flagella are probably controlled by signaling processes inside the cell.

We tracked the waveforms of each flagellum using the GVF technique^38,39^ with high spatio-temporal resolution, allowing us to characterize the bending waves that propagate from basal ends towards the distal tips at a frequency of 25 ± 4 Hz. We performed mode analysis of flagella exhibiting rhythmic patterns^40^ to define a continuous phase for each oscillator flagellum. The phase analysis captures the dynamics of two interacting flagella in basal apparatus, confirming that they effectively act as two isolated pendulums perturbing each other by non-linear fluctuations, but no entrainment can occur and interflagellar phase difference grows linearly over time with a temporal slope of Δ*f*.^19,26^

We used our tracked data to examine the validity of resistive force theory for flagellar apparatus swimming effectively in 2D. From experimental recorded videos, we extracted the position of the basal body with sub-micron resolution and calculated the translational and rotational velocities. It is to be noted that as flagellar apparatus swims, the angle of the V junction changes over time (see Videos S1 and S2, ESI†). We computed the instantaneous V-angle from the tracked data and used it as an experimental input for our RFT analysis. Comparing our experimental data with instantaneous translational and rotational velocities of basal apparatus obtained by RFT simulations, we found a quantitative agreement with a drag anisotropy of 2. Originally, the ratio *ζ*_⊥_/*ζ*_‖_ = 2 was used by Gray and Hancock for sea-urchin spermatozoa swimming far away from the boundary.^52^ In our experiments, the flagellar apparatus swims in the vicinity of a glass surface and in this respect, our system is similar to experiments with microtubules moving parallel to a kinesin-coated substrate which can exert forces on microtubules.^53^

To investigate the swimming dynamics, we performed simulations and analytical approximations using simplified wave forms, allowing us to estimate the mean rotational velocity of flagellar apparatus in the limit of small intrinsic curvature and wave amplitude. To calculate the forces excreted by the fluid on cylindrical segments of each flagellum we used RFT which ignores long-range hydrodynamic interactions between different parts of the flagella. Interestingly, our analysis demonstrates that by introducing a phase or frequency difference between two flagella, steering of flagellar apparatus can occur.

The results presented in this study are focused on the examples where the basal apparatus swims effectively in 2D in the vicinity of the substrate. This greatly facilitates the tracking of flagella and data analysis. However, we observed several examples (*N* = 8) where basal apparatus swims in 3D and undergoes tumbling motion, as shown in the Videos S16 and S17 (ESI†). This out of plane swimming dynamics complicates the tracking process of two flagella. In future studies, 3D microscopy techniques^54^ are necessary to capture the full swimming dynamics of basal apparatus. In addition, we observed often in our experiments basal apparatus with only one beating flagellum while the second one is either not active or the activity is very low (see Videos S18–S20, ESI†). Therefore, not all isolated basal apparatus are suitable for synchronization analysis, which further reduces the yield of the experiments. Furthermore, we emphasize that our experiments are performed with one specific strain of *Chlamydomonas* cells and experiments with other strains are necessary to check the generality of our results on synchronization dynamics.

It would be worthwhile to extend our analysis to flagellar apparatus without intrinsic frequency mismatch as experimentally observed in ref. 35 and 36. Lastly, Hyams and Borisy reported interesting observations of switching the swimming direction of basal apparatus from forward to backward motion at calcium concentrations above 1 μM. In our experiments, under standard reactivation conditions, only forward swimming motion was observed. Calcium ions presumably affect the form and direction of ciliary beating patterns and it is known that exchange of calcium ions is crucial for tactic behavior of *C. reinhardtii* cells.^36,55^ Investigations in this direction are under way in our laboratory.

## Author contributions

A. G. designed the research. A. G. and S. G. P. performed the experiments. A. B. wrote the Matlab code for GVF algorithm. S. G. P. did the tracking of the flagellar apparatus. A. G. and A. B. performed RFT analysis, simulations and analytical calculations. A. G. wrote the first draft of the manuscript and all the authors contributed to the discussions and the final version of the manuscript.

## Conflicts of interest

There are no conflicts to declare.

## Supplementary Material

SM-017-D0SM01969K-s001

SM-017-D0SM01969K-s002

SM-017-D0SM01969K-s003

SM-017-D0SM01969K-s004

SM-017-D0SM01969K-s005

SM-017-D0SM01969K-s006

SM-017-D0SM01969K-s007

SM-017-D0SM01969K-s008

SM-017-D0SM01969K-s009

SM-017-D0SM01969K-s010

SM-017-D0SM01969K-s011

SM-017-D0SM01969K-s012

SM-017-D0SM01969K-s013

SM-017-D0SM01969K-s014

SM-017-D0SM01969K-s015

SM-017-D0SM01969K-s016

SM-017-D0SM01969K-s017

SM-017-D0SM01969K-s018

SM-017-D0SM01969K-s019

SM-017-D0SM01969K-s020

SM-017-D0SM01969K-s021

SM-017-D0SM01969K-s022

SM-017-D0SM01969K-s023

SM-017-D0SM01969K-s024

SM-017-D0SM01969K-s025

SM-017-D0SM01969K-s026

SM-017-D0SM01969K-s027
