# Supplementary figures and images for: Resistive force theory and wave dynamics in swimming flagellar apparatus isolated from C. reinhardtii
Source: Soft Matter. 2020 Dec 9;17(6):1601–13. doi: 10.1039/d0sm01969k (PMC8323821; doi:10.1039/d0sm01969k)

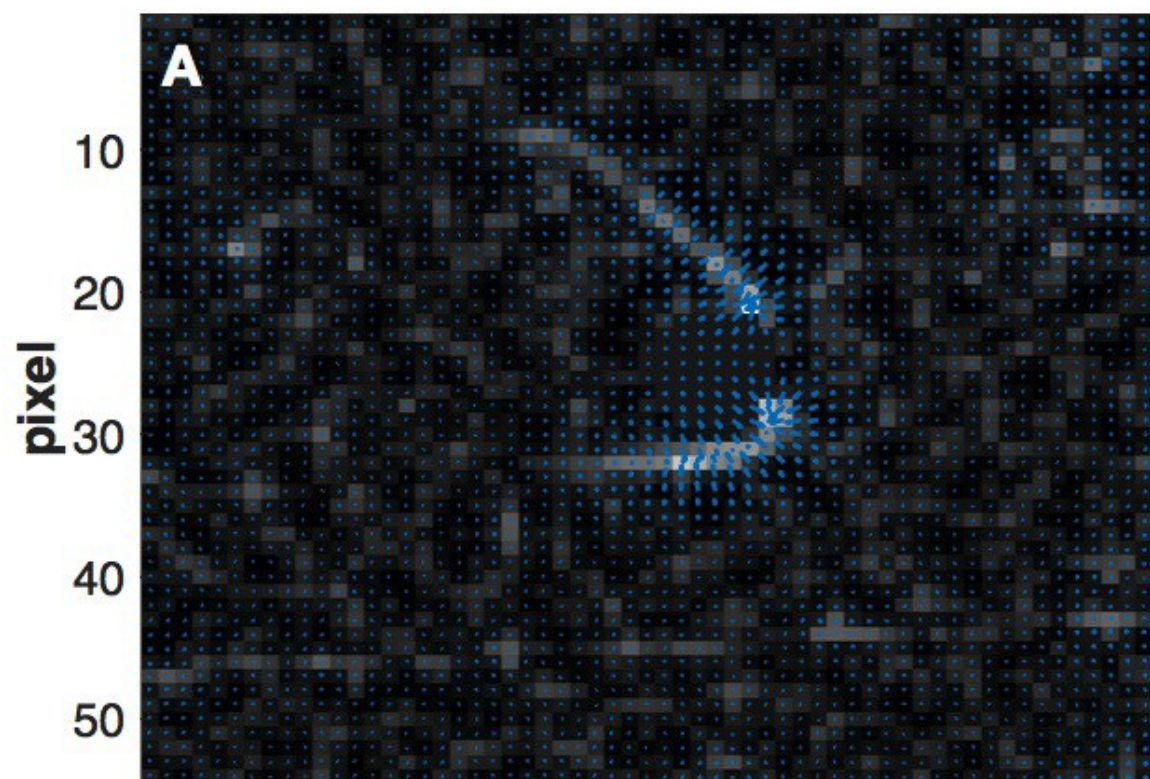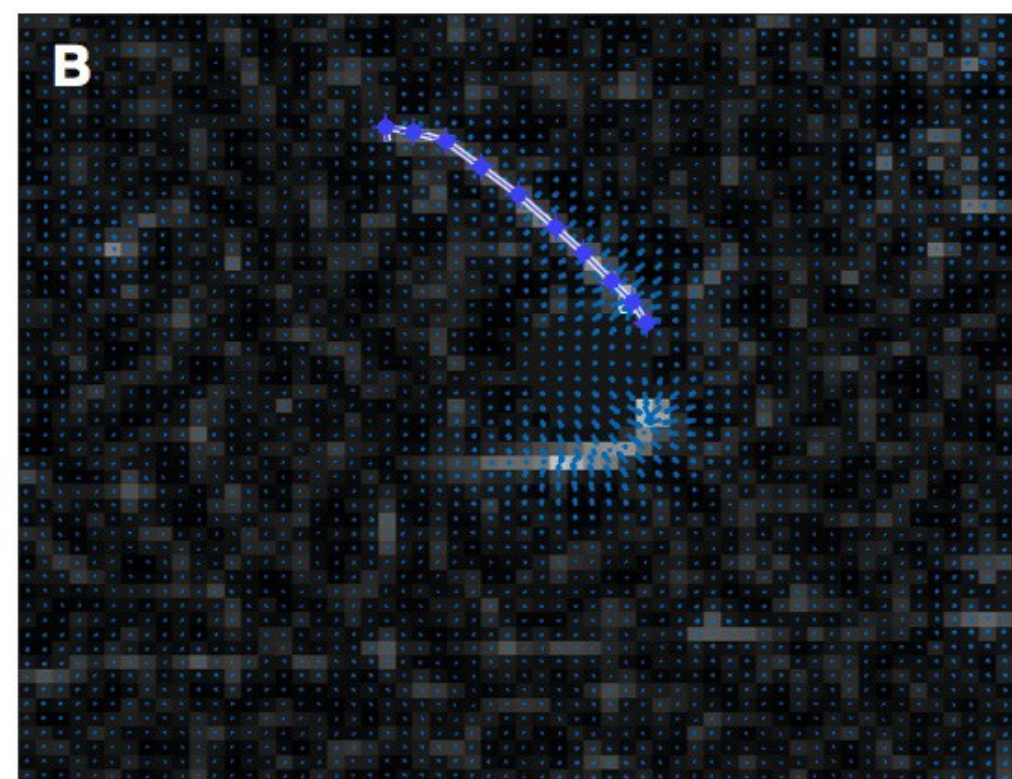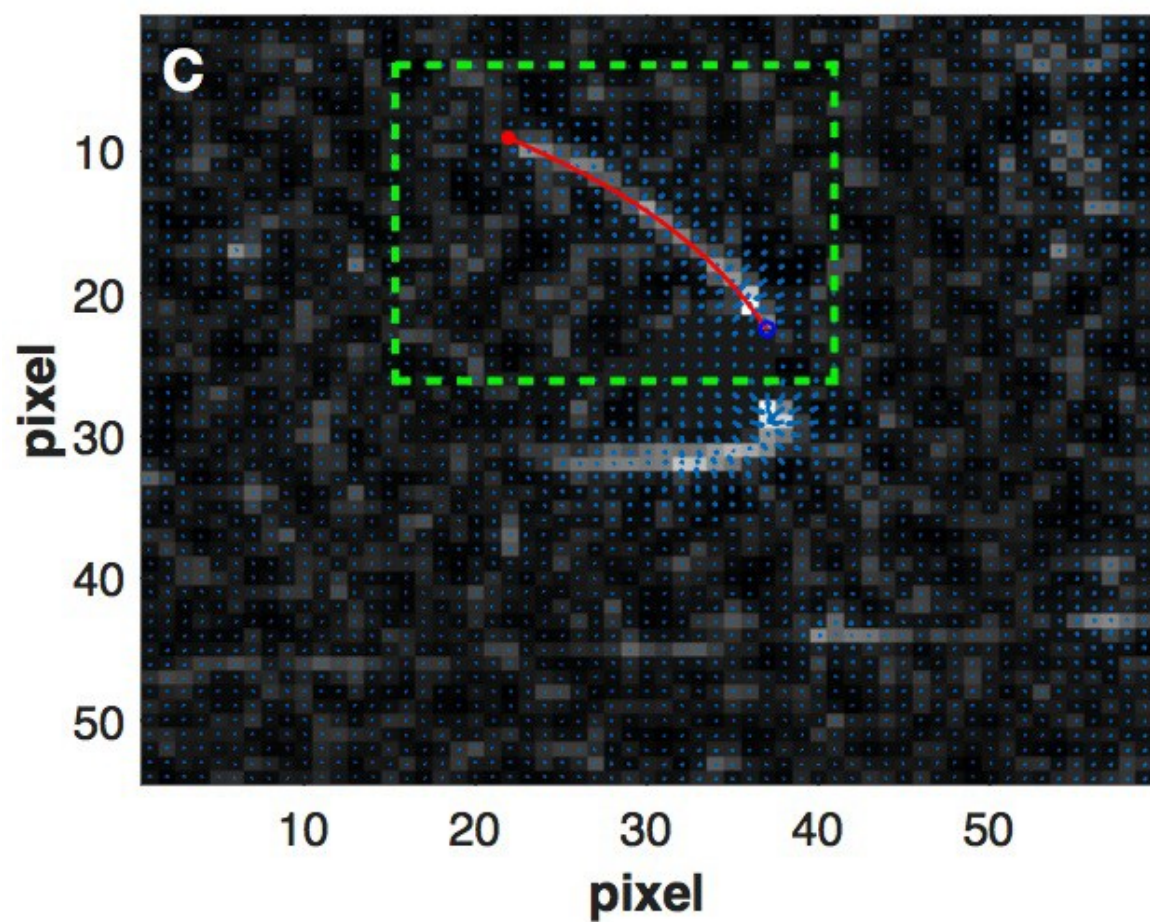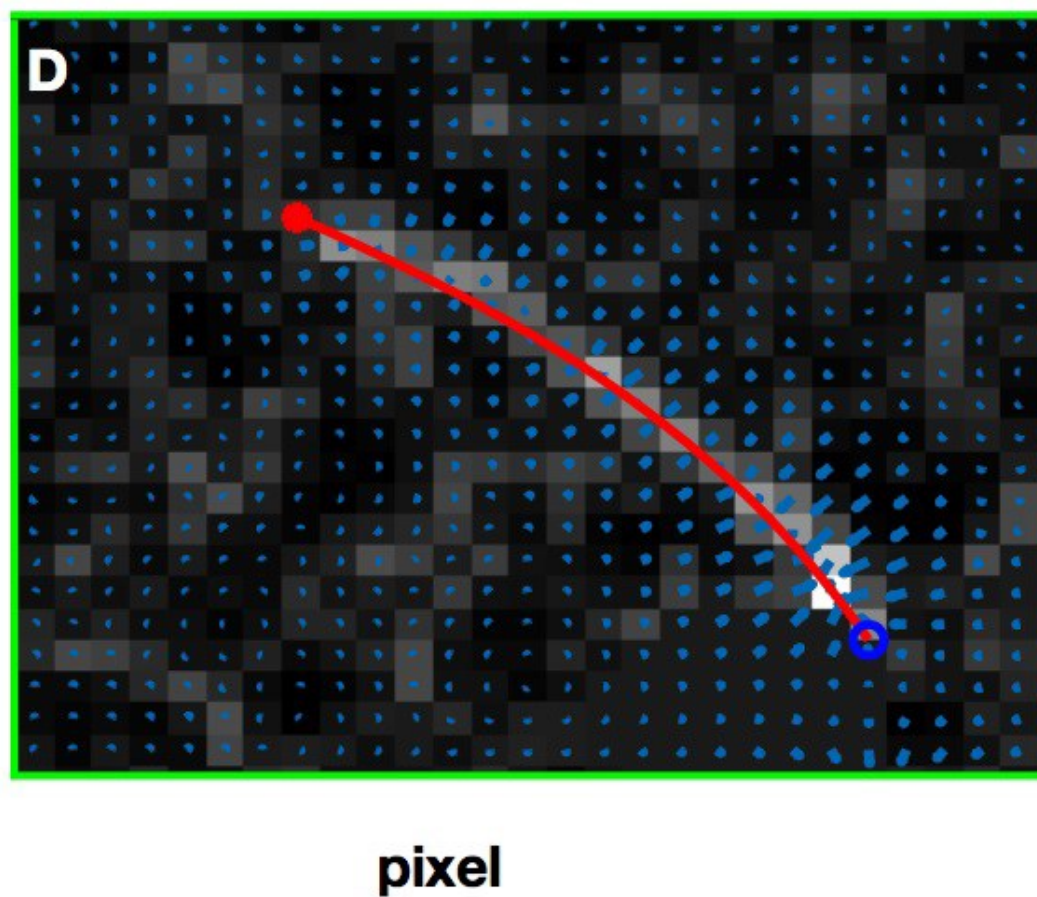

Supplement: SM-017-D0SM01969K-s012 [file SM-017-D0SM01969K-s012.pdf]

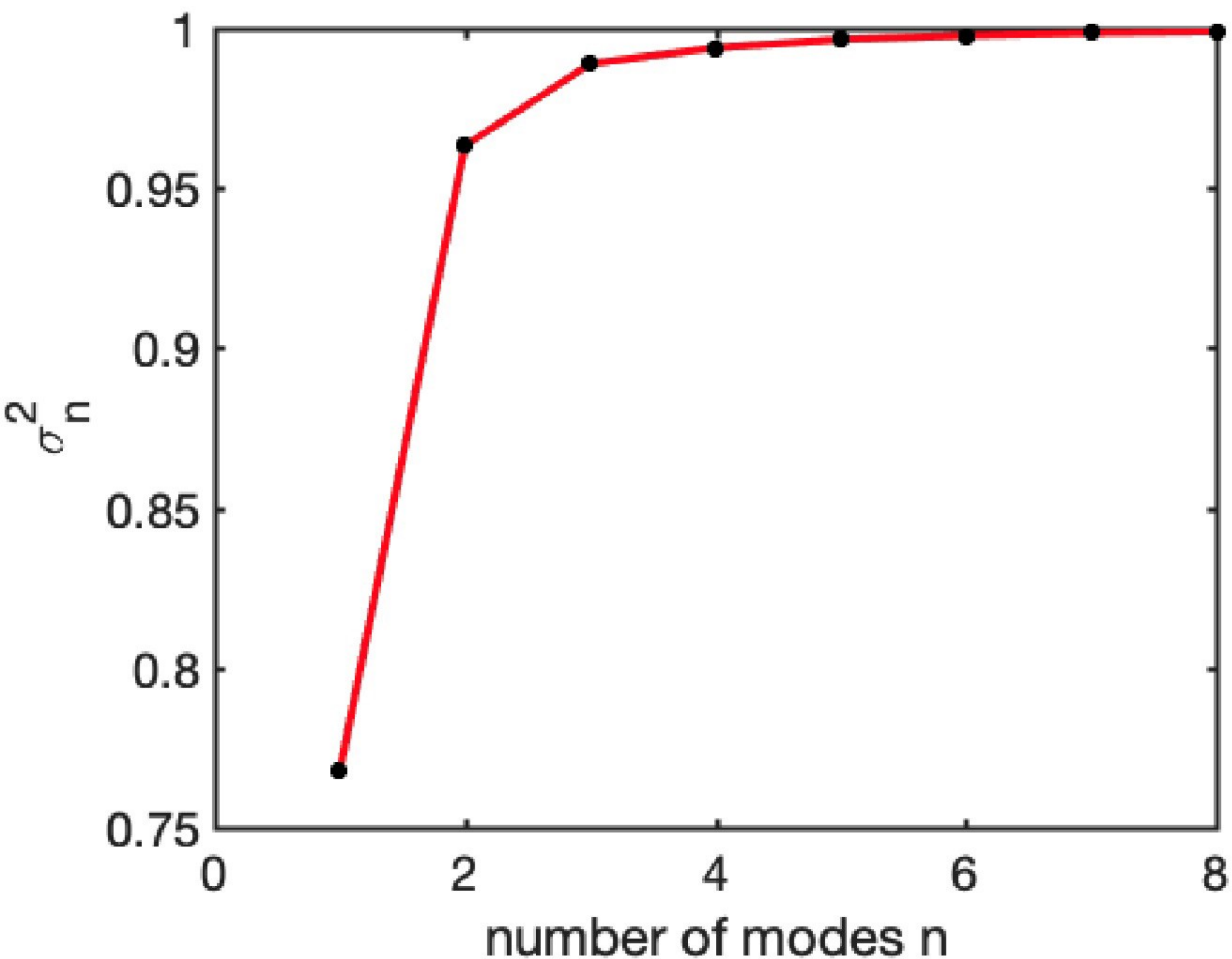

Supplement: SM-017-D0SM01969K-s021 [file SM-017-D0SM01969K-s021.pdf]

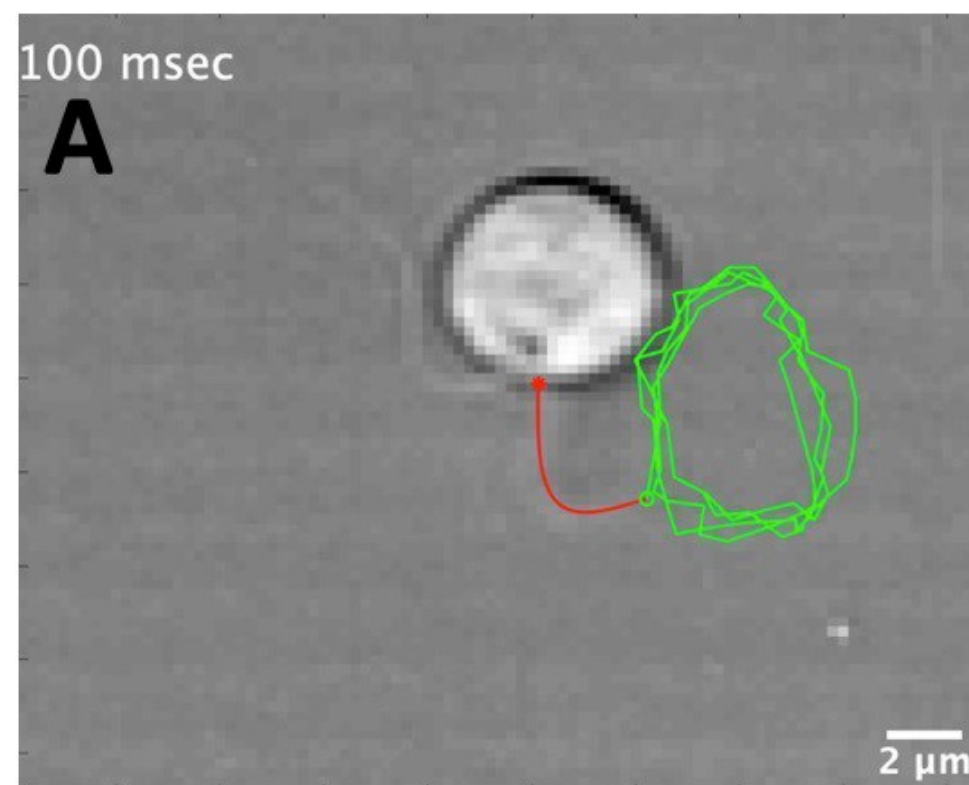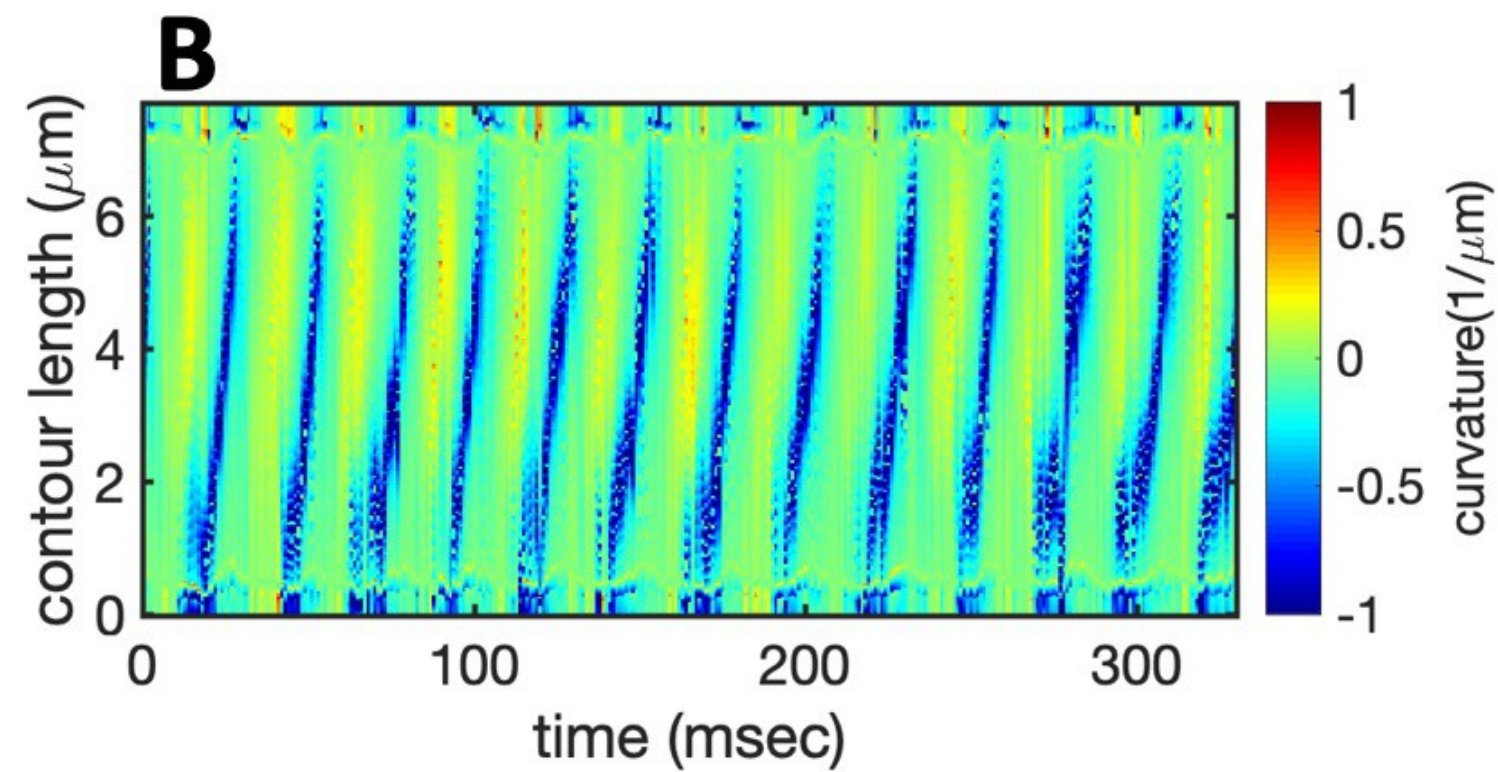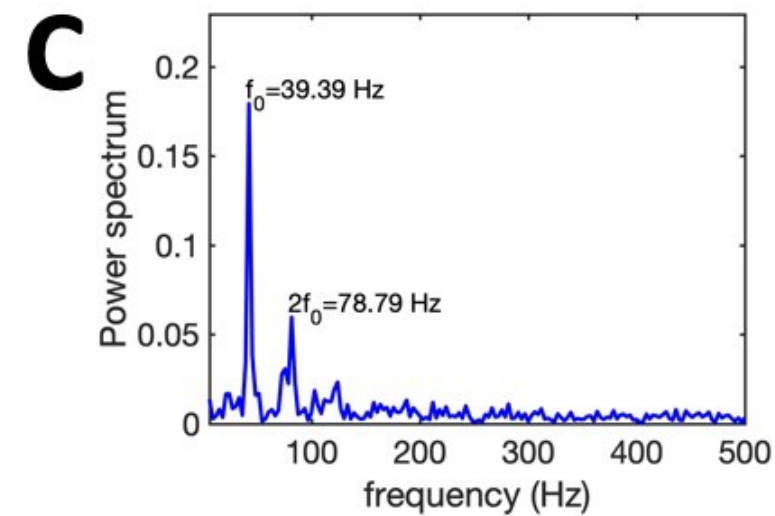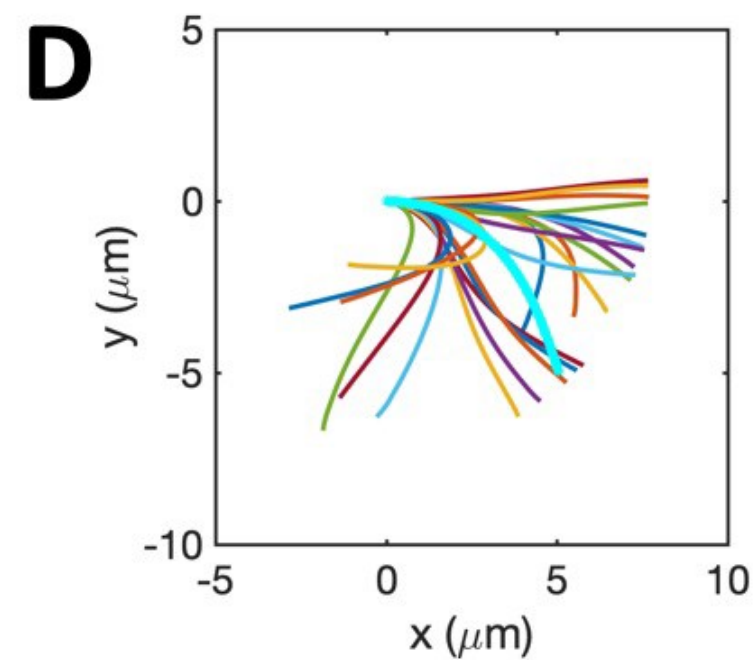

Supplement: SM-017-D0SM01969K-s022 [file SM-017-D0SM01969K-s022.pdf]

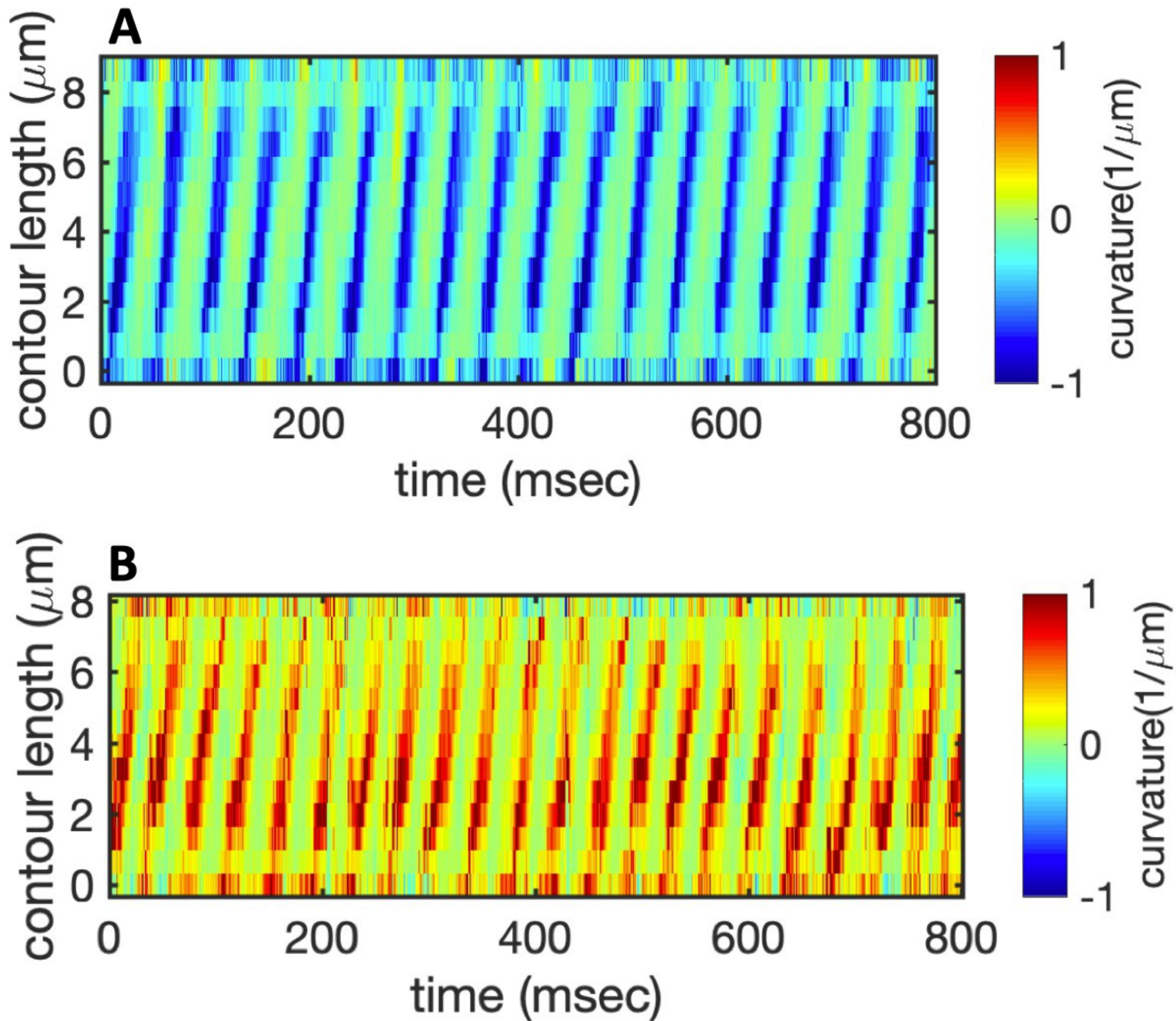

Supplement: SM-017-D0SM01969K-s023 [file SM-017-D0SM01969K-s023.pdf]

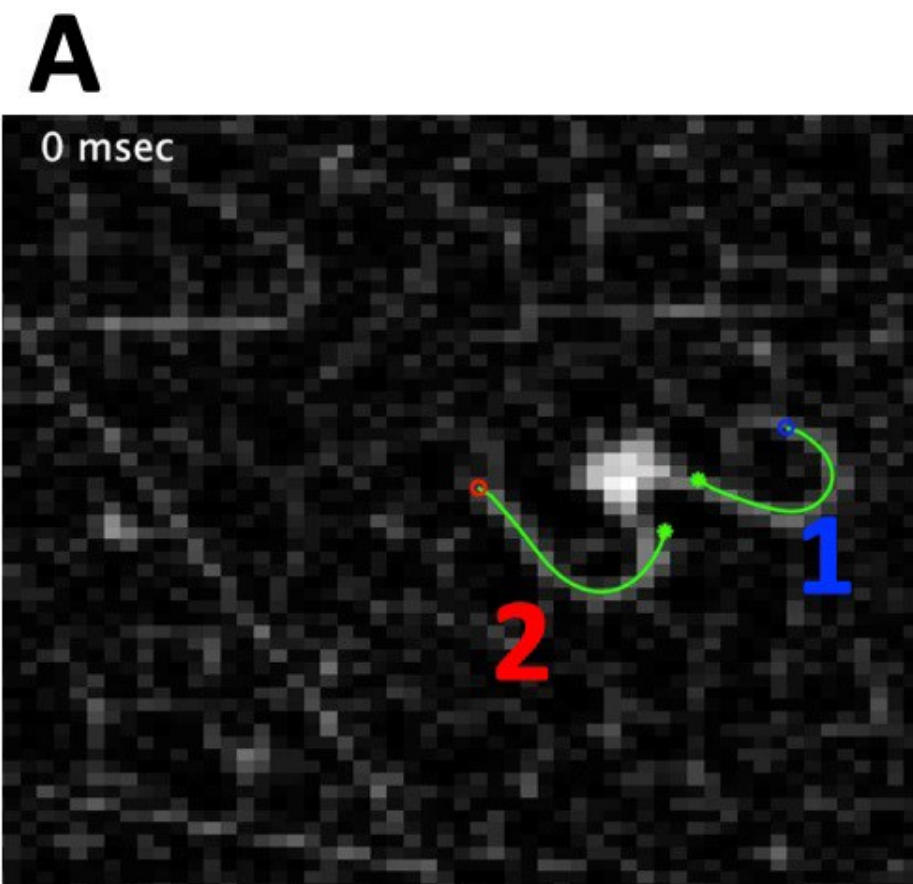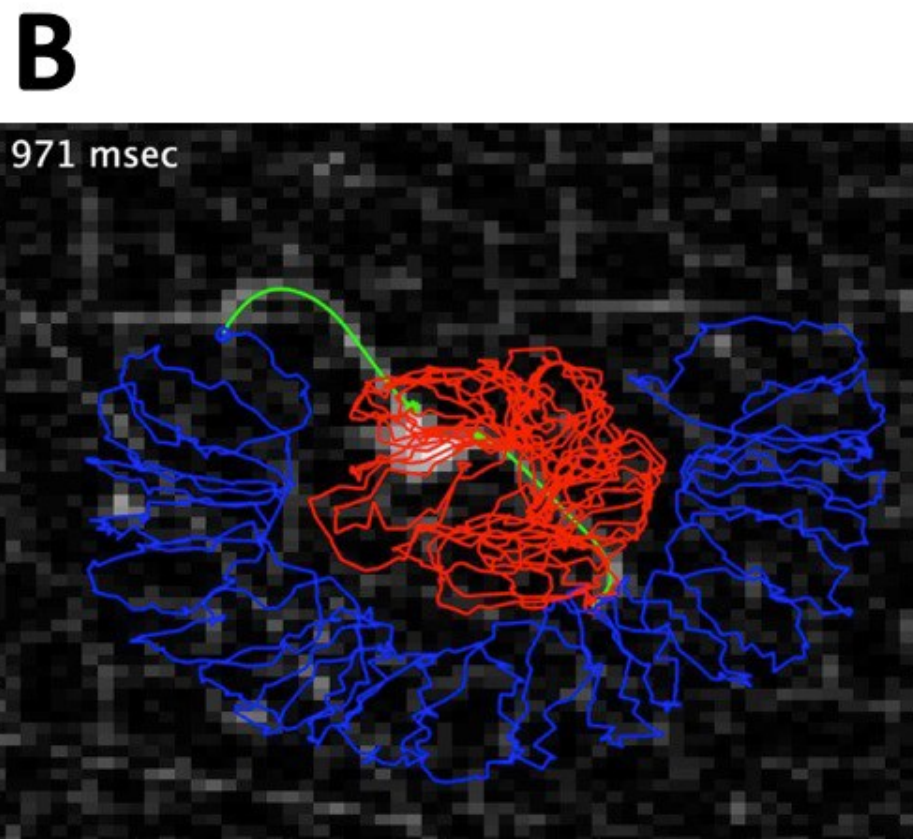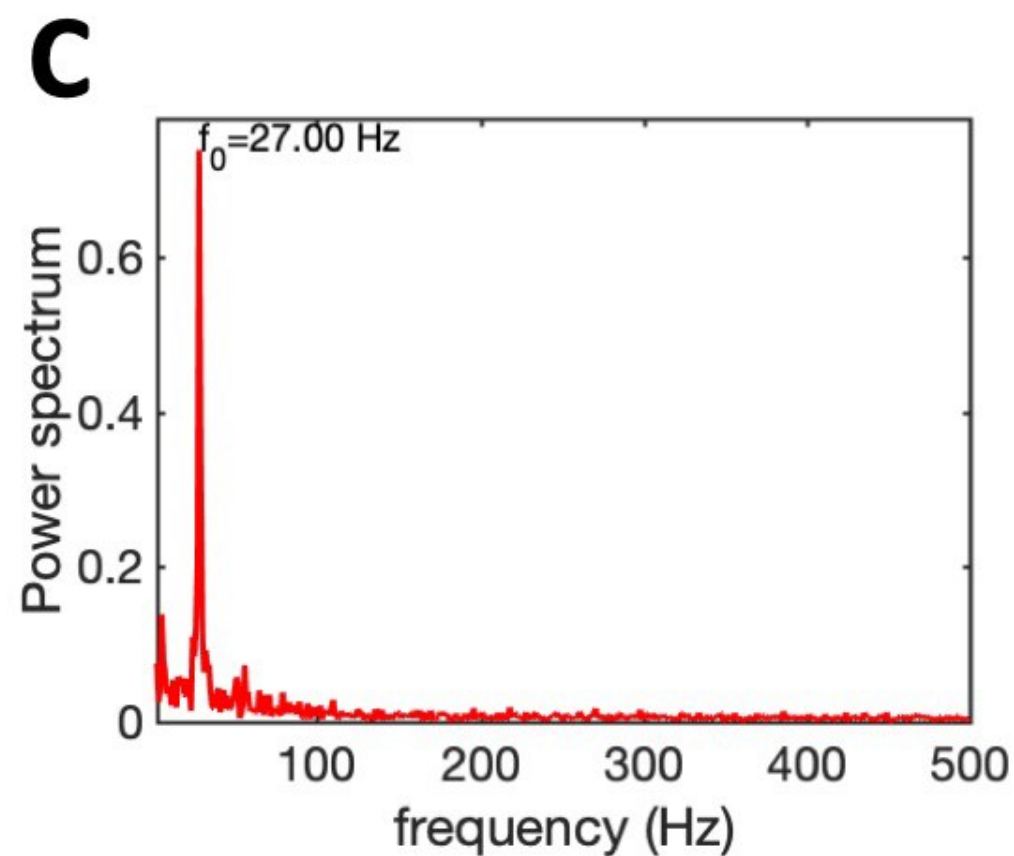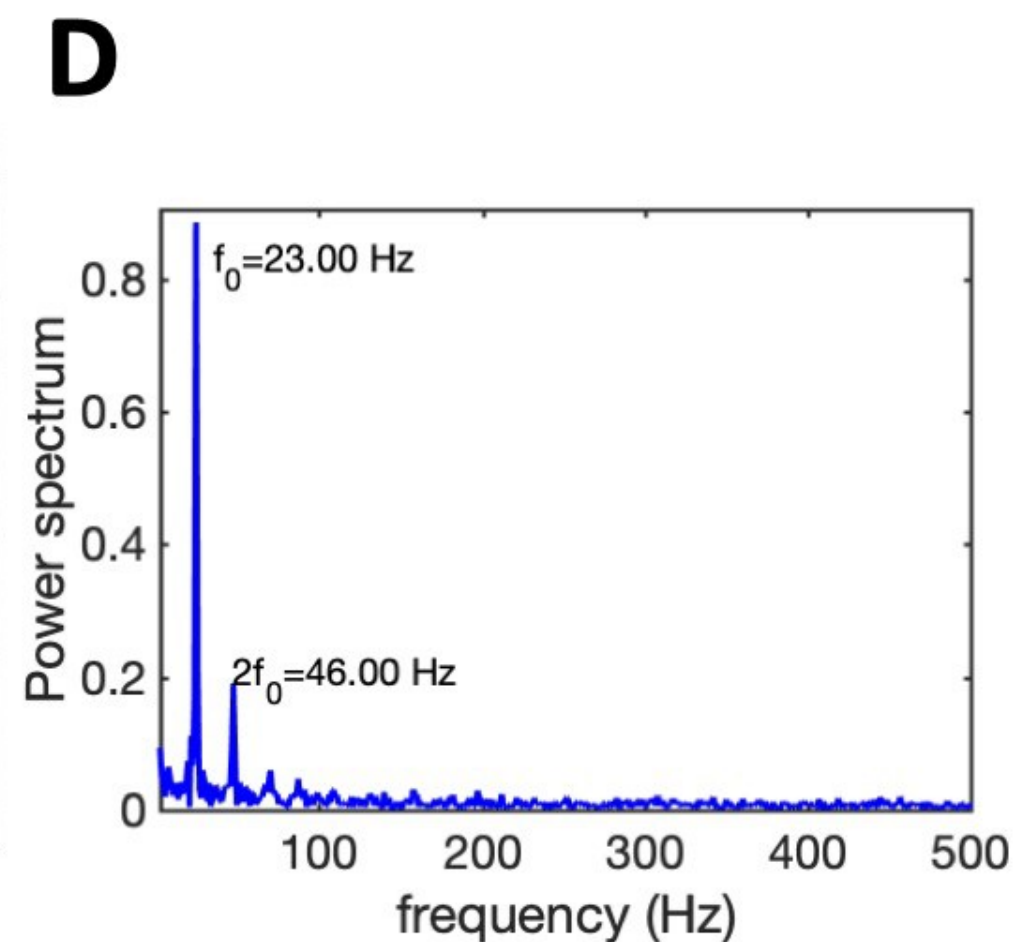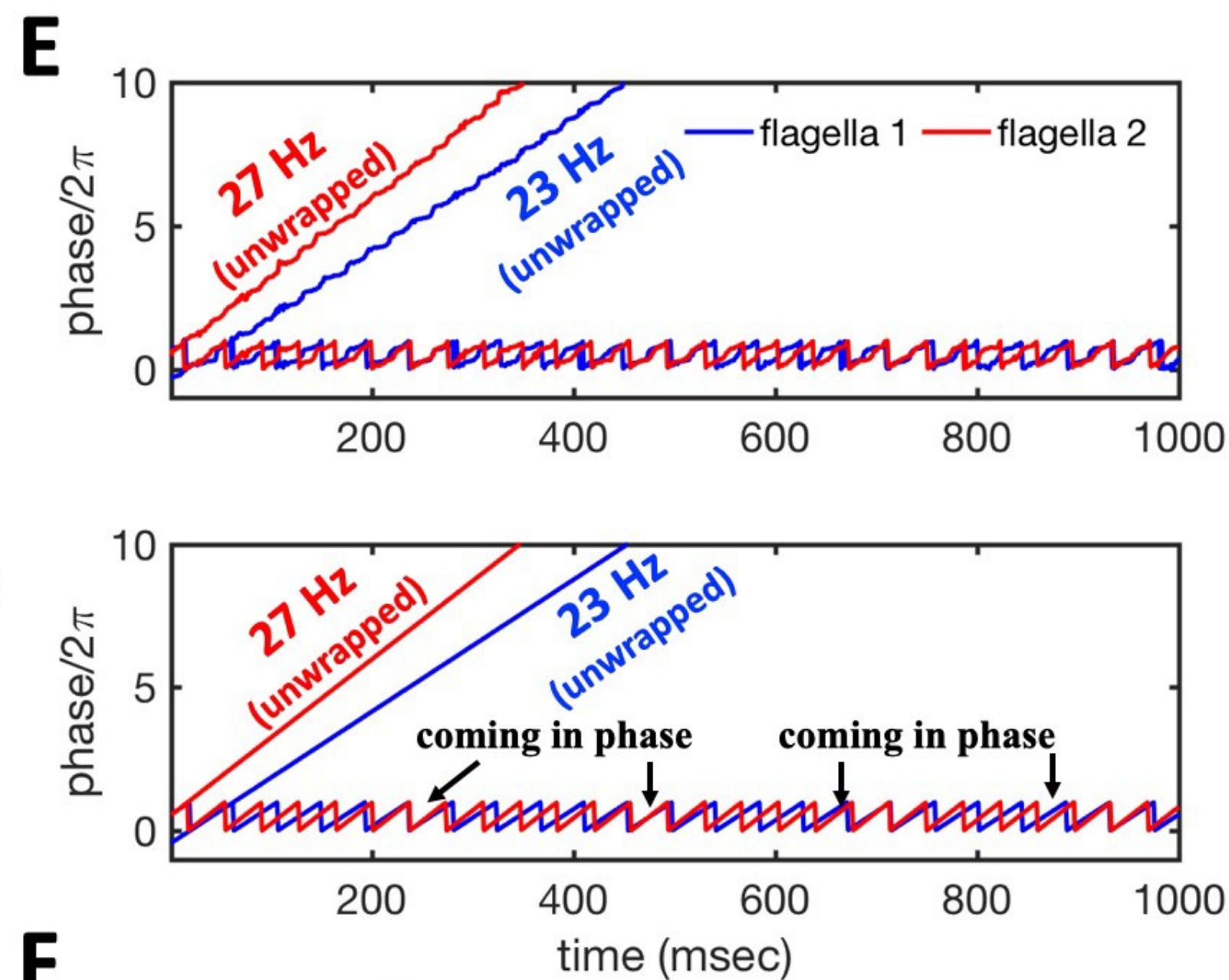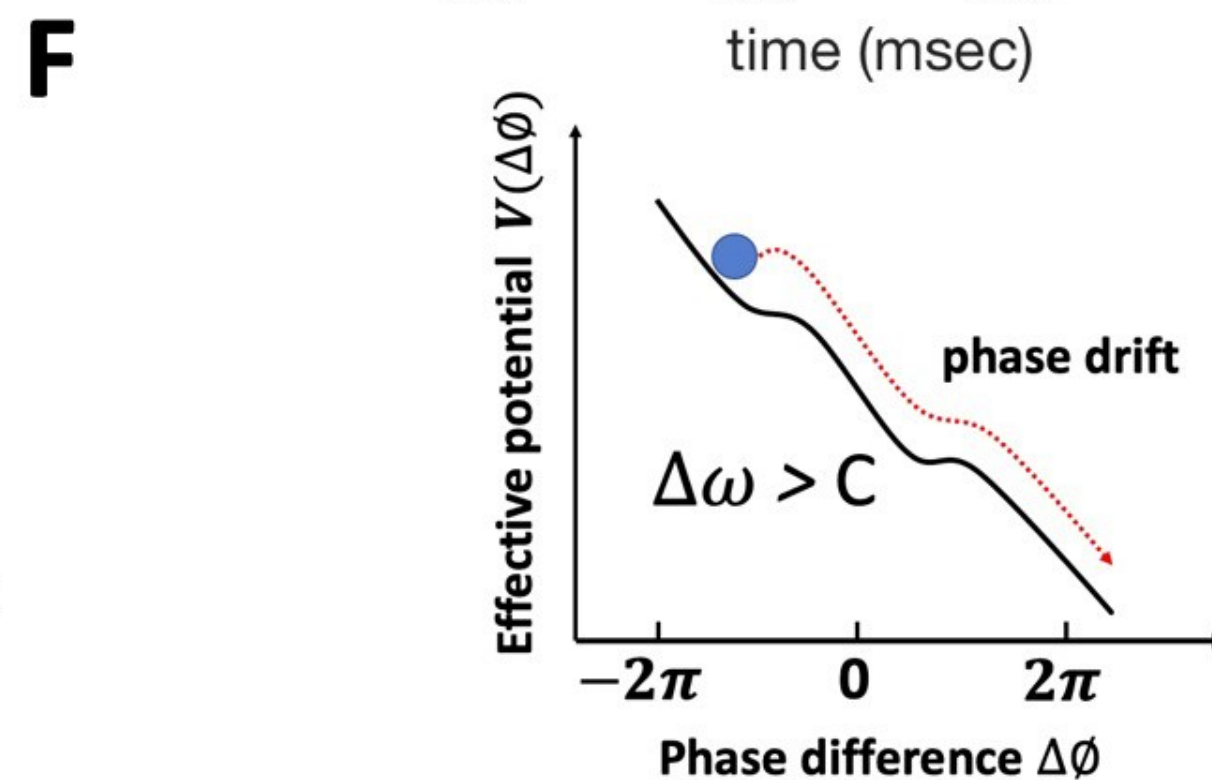

Supplement: SM-017-D0SM01969K-s024 [file SM-017-D0SM01969K-s024.pdf]
