# Supplementary material for: Resistive force theory and wave dynamics in swimming flagellar apparatus isolated from C. reinhardtii
Source: Soft Matter. 2020 Dec 9;17(6):1601–13. doi: 10.1039/d0sm01969k (PMC8323821; doi:10.1039/d0sm01969k)
Supplement: SM-017-D0SM01969K-s025 [file SM-017-D0SM01969K-s025.pdf]

Electronic Supplementary Material (ESI) for Soft Matter.
This journal is © The Royal Society of Chemistry 2021

Soft Matter

ARTICLE TYPE

Cite this: DOI: 00.0000/xxxxxxxxxx

Resistive force theory and wave dynamics in swimming flagellar apparatus isolated from C. reinhardtii†

Samira Goli Pozveh, Albert J Bae,‡ and Azam Gholami*

Supplemental Information

Approximation of mean rotational velocity

First, we consider the dynamics of a single flagellum. In the simplified form, curvature waves propagating along the contour length of a flagellum is given by:

$$\kappa(s,t) = \kappa_0 + \kappa_1 \cos[(ks - \omega t + 2\pi\alpha)], \tag{S.1}$$

where $\omega = 2\pi f_0$ and $k = 2\pi/\lambda \sim 2\pi/L$. Tangential angle θ is computed as:

$$\theta(s,t) = \int_0^s ds' \kappa(s',t) = \kappa_0 s + \frac{\kappa_1 L}{2\pi}\Big(\sin[2\pi(s/L - f_0 t + \alpha)] - \sin[2\pi(-f_0 t + \alpha)]\Big) + \theta(0,t). \tag{S.2}$$

For simplicity, we assume flagella is clamped at $s = 0$ along the $\hat{x}$-axis, i.e. $\theta(0,t) = 0$ and $x(s = 0,t) = y(s = 0,t) = 0$. Assuming both $\kappa_0/k \sim \kappa_0 L/(2\pi)$ and $\kappa_1/k \sim \kappa_1 L/(2\pi)$ to be small, we approximate $\cos\theta$ and $\sin\theta$ by keeping $\kappa_0 L/(2\pi)$ up to the second order and $\kappa_1 L/(2\pi)$ up to the third order:

$$\cos[\theta(s,t)] \approx 1 - \frac{\kappa_0^2 s^2}{2} + \frac{1}{24\pi^3}\kappa_1 L\cos[\pi(2\alpha + \frac{s}{L} - 2f_0 t)]\sin[\frac{\pi s}{L}] \Bigg(-24\kappa_0\pi^2 s + 2\kappa_1 L\cos[\pi(2\alpha + \frac{s}{L} - 2ft)]\sin[\frac{\pi s}{L}]\Big(3\pi(-2 + \kappa_0^2 s^2) + \kappa_0\kappa_1 Ls\Big(-\sin[2\pi(\alpha - f_0 t)] + \sin[\frac{2\pi(\alpha L + s - f_0 L t)}{L}]\Big)\Big)\Bigg), \tag{S.3}$$

$$\sin[\theta(s,t)] \approx \kappa_0 s + \frac{1}{12\pi^3}\kappa_1 L\cos[\pi(2\alpha + \frac{s}{L} - 2f_0 t)]\sin[\frac{\pi s}{L}]\Bigg(-6\pi^2(-2 + \kappa_0^2 s^2) + \kappa_1 L\cos[\pi(2\alpha + \frac{s}{L} - 2f_0 t)]\sin[\frac{\pi s}{L}]\Big(-6\kappa_0\pi s + \kappa_1 L(-2 + \kappa_0^2 s^2) \cos[\pi(2\alpha + \frac{s}{L} - 2f_0 t)]\sin[\frac{\pi s}{L}]\Big)\Bigg). \tag{S.4}$$

Max Planck Institute for Dynamics and Self-Organization, Göttingen, Germany. E-mail: azam.gholami@ds.mpg.de

† Electronic Supplementary Information (ESI) available: See DOI: 00.0000/00000000.

‡ Current address: Department of Biomedical Engineering, University of Rochester, USA

Flagella's centerline is described by vector $\mathbf{r}(s,t) = (\int_0^s ds' \cos[\theta(s',t)], \int_0^s ds' \sin[\theta(s',t)])$ and local velocity and unit tangent vectors are calculated as $\mathbf{U}(s,t) = (dx/dt, dy/dt)$ and $\mathbf{t} = (\mathbf{t}_x, \mathbf{t}_y) = (\cos[\theta(s,t)], \sin[\theta(s,t)])$, respectively.

We calculate instantaneous active torque around the grafting point at $(0,0)$ as:

$$\tau_a(t) = \int_0^L ds\, \mathbf{r}(s,t) \times \mathbf{f}(s,t), \tag{S.5}$$

where we $\mathbf{f}(s,t)$ is computed in the framework of resistive force theory:

$$\mathbf{f}(s,t) = -[\zeta_\parallel \mathbf{tt} + \zeta_\perp(\mathbf{I} - \mathbf{tt})].\mathbf{U} = -\zeta_\perp(\mathbf{I} - (1-\eta)\mathbf{tt}).\mathbf{U}. \tag{S.6}$$

Here $\mathbf{tt} + \mathbf{nn} = \mathbf{I}$ is the identity matrix and $1 - \eta = 1 - \zeta_\parallel/\zeta_\perp$ is a measure of anisotropy in drag coefficients. Note that if we calculate time-average of force $\mathbf{f}(s,t)$, the first term in Eq. S.6 vanishes and only the second term which is proportional to drag anisotropy contributes in the mean force. However, the first term does not vanish when we calculate the mean torque. Thus, $\tau_a(t)$ has a term proportional to $\zeta_\perp$ and a second term proportional to $\zeta_\perp(1-\eta)$:

$$\tau_a(t) = -\zeta_\perp \int_0^L ds\, [\mathbf{r}(s,t) \times \mathbf{U}(s,t)] + (1-\eta)\zeta_\perp \int_0^L ds\, [(\mathbf{r}(s,t) \times \mathbf{t}(s,t))][\mathbf{t}(s,t).\mathbf{U}(s,t)]. \tag{S.7}$$

For a planar flagellar beat in $x-y$ plane, $\tau_a(t)$ is in $\hat{z}$ direction. In the limit of $\lambda \to L$ and up to first order in $\kappa_0 L/(2\pi)$ and second

order in $\kappa_1 L/(2\pi)$, we obtain:

$$\tau_a \approx \frac{\zeta_\perp f_0 \kappa_1 L^4}{829440\pi^6}\Bigg(-27\Big(80\pi^2\big(32\pi^2\ (3+4\pi^2)+\kappa_1^2 L^2(-60+75\eta + 16(-2+\eta)\pi^2)\big)+\kappa_0^2 L^2\big(\kappa_1^2 L^2(315(-23+30\eta)-2940(-1+\eta)\pi^2 + 64(11-10\eta)\pi^4)+128\pi^2(15(2+\eta)-10(-1+\eta)\pi^2+4(-4+3\eta)\pi^4)\big)\Big) \times \cos[2\pi(\alpha-f_0 t)]+5\Big(1728\kappa_0\kappa_1 L^2\pi^2\big(15-22\pi^2+9\eta(-5+2\pi^2)\big) + 648\kappa_0\kappa_1 L^2\pi^2\big(15-48\pi^2+5\eta(-9+8\pi^2)\big)\cos[4\pi(\alpha-f_0 t)] + \kappa_1^2 L^2\big(720(1-3\eta)\pi^2+\kappa_0^2 L^2(2275-3745\eta+4164(-1+\eta)\pi^2)\big) \cos[6\pi(\alpha-f_0 t)]-108\pi\big(24(-3+\eta)\kappa_1^2 L^2\pi^2+384\pi^4+\kappa_0^2 L^2(96(2+\eta)\pi^2 + 32(-4+3\eta)\pi^4+\kappa_1^2 L^2(-27+12\eta+30\pi^2-26\eta\pi^2))\big)\sin[2\pi(\alpha-f_0 t)] + 1296(3-17\eta)\kappa_0\kappa_1 L^2\pi^3\sin[4\pi(\alpha-f_0 t)]-6\kappa_1^2 L^2\pi\big(144(2-3\eta)\pi^2 + \kappa_0^2 L^2(-44+185\eta+12(-17+18\eta)\pi^2)\big)\sin[6\pi(\alpha-f_0 t)]\Big)\Bigg) \tag{S.8}$$

As mentioned previously, even in the absence of drag anisotropy ($\eta = \zeta_\parallel/\zeta_\perp = 1$), Eq. S.8 has a non-zero value proportional to $\zeta_\perp$. In parallel, we approximate the instantaneous viscous torque exerted on a flagellum which deforms its shape over time while it is pinned at one end and rotates at instantaneous angular velocity $\Omega = (0,0,\Omega_z(\kappa_0,\kappa_1,f_0,\alpha,\eta,t))$ around the pinning point. At any instant of time, we consider flagella apparatus as a solid body with rotational velocity Ω_z and calculate the viscous torque as:

$$\begin{aligned}\tau_v(\kappa_0,\kappa_1,f_0,\alpha,\eta,\zeta_\perp,t) &= -\int_0^L ds\ \mathbf{r}(s,t)\times\mathbf{f}(s,t) \\ &= D(\kappa_0,\kappa_1,f_0,\alpha,\eta,\zeta_\perp,t)\Omega_z\end{aligned} \tag{S.9}$$

where $\mathbf{f}(s,t)$ is calculated using Eq. S.6 with instantaneous rigid body velocity $\mathbf{U} = \Omega\times\mathbf{r}(s,t) = [-y(s,t)\hat{x}+x(s,t)\hat{y}]\Omega_z$ and D is the instantaneous drag coefficient that we aim to calculate. After performing the integration in Eq. S.9, we obtain instantaneous drag coefficient as:

$$D \approx \frac{\zeta_\perp L^3}{829440\pi^6}\Bigg(864\Big(-320\pi^6-20\kappa_1^2 L^2\pi^2\big(-3-4\pi^2+2\eta(3+\pi^2)\big) + \kappa_0^2 L^2(16(4-3\eta)\pi^6+\kappa_1^2 L^2(45(3-5\eta)+90(-1+\eta)\pi^2+ 2(-11+10\eta)\pi^4))\Big)+5\kappa_1 L^2\Big(54\kappa_0\big(3\kappa_1^2 L^2\big(-45+75\eta+112\pi^2-104\eta\pi^2\big) + 256\pi^2(3-6\eta-5\pi^2+4\eta\pi^2)\big)\cos[2\pi(\alpha-f_0 t)]+243\kappa_1\big(16(1-2\eta)\pi^2 + 5\kappa_0^2 L^2(8-11\eta+8(-1+\eta)\pi^2)\big)\cos[4\pi(\alpha-f_0 t)] + 2\kappa_0\kappa_1^2 L^2\Big(395-336\pi^2+\eta(-565+312\pi^2)\Big)\cos[6\pi(\alpha-f_0 t)] - 324\kappa_0\pi\big(3(13-17\eta)\kappa_1^2 L^2+128(-1+2\eta)\pi^2\big)\sin[2\pi(\alpha-f_0 t)] - 54\kappa_1\pi\Big(48(1-2\eta)\pi^2+\kappa_0^2 L^2\big(-40(3+\pi^2)+\eta(159+44\pi^2)\big)\Big)\times \sin[4\pi(\alpha-f_0 t)]+12(-5+\eta)\kappa_0\kappa_1^2 L^2\pi\sin[6\pi(\alpha-f_0 t)]\Big)\Bigg) \tag{S.10}$$

Note that for $\kappa_0 = \kappa_1 = 0$, Eq. S.10 reduces to $-\zeta_\perp L^3/3$ which is the drag coefficient of a rigid cylinder of length L. Furthermore, in the limit of $\kappa_1 = 0$, Eq. S.10 simplifies to $-\zeta_\perp L^3\big(20+\kappa_0^2(-4+3\eta)L^2\big)/60$ which is the drag coefficient of a bent cylinder with mean curvature κ_0.

Now, we can estimate mean rotational velocity for a single flagellum as:

$$\langle\Omega_z(\kappa_0,\kappa_1,f_0,\eta)\rangle = f_0\int_0^{1/f_0} dt\ \frac{\tau_a(\kappa_0,\kappa_1,f_0,\alpha,t)}{D(\kappa_0,\kappa_1,f_0,\alpha,t)}. \tag{S.11}$$

Before performing the integration over time, we expand the ratio of τ_a/D up to first order in $\kappa_0 L/(2\pi)$ and second order in $\kappa_1 L/(2\pi)$, to obtain:

$$\langle\Omega_z\rangle \approx -f_0\frac{\kappa_0\kappa_1^2 L^3}{32\pi^6}(2\pi^2-3)\Big((\pi^2-3)-\eta(\pi^2-6)\Big) \tag{S.12}$$

As expected, by integrating over one beat cycle, α averages out. Moreover, consistent with our simulations, $\langle\Omega_z\rangle$ is non-zero for isotropic drag coefficients $\zeta_\parallel = \zeta_\perp$.

After estimating τ_a, τ_v and $\langle\Omega_z\rangle$ for a single flagellum, we can now calculate instantaneous rotational velocity Ω_z of a flagellar apparatus with two flagella, ignoring the viscous drag of basal body for simplicity. We balance instantaneous total active torque with instantaneous total drag torque exerted on flagella apparatus to obtain:

$$\Omega_z(\kappa_0,\kappa_1,\kappa_0',\kappa_1',\alpha,f_0,\eta,t) = \frac{\tau_a(\kappa_0,\kappa_1,f_0,0,t)+\tau_a(\kappa_0',\kappa_1',f_0,\alpha,t)}{D(\kappa_0,\kappa_1,f_0,0,t)+D(\kappa_0',\kappa_1',f_0,\alpha,t)} \tag{S.13}$$

Note that two flagella have different values of intrinsic and dynamic curvature and beat with a phase difference of $2\pi\alpha$. Next, to estimate time-averaged rotational velocity $\langle\Omega_z\rangle$ of flagellar apparatus, we integrate over one beating cycle:

$$\langle\Omega_z\rangle = f_0\int_0^{1/f_0} dt\ \frac{\tau_a(\kappa_0,\kappa_1,f_0,0,t)+\tau_a(\kappa_0',\kappa_1',f_0,\alpha,t)}{D(\kappa_0,\kappa_1,f_0,0,t)+D(\kappa_0',\kappa_1',f_0,\alpha,t)} \tag{S.14}$$

Here, for simplicity we have assumed that both flagella beat at the same frequency f_0. Before doing the integration, we expand the ratio of total active torque to instantaneous drag coefficients up to first order in $\kappa_0 L/(2\pi)$ and $\kappa_0' L/(2\pi)$ but second order in $\kappa_1 L/(2\pi)$ and $\kappa_1' L/(2\pi)$ to obtain:

$$\langle\Omega_z\rangle \propto \frac{f_0 L^3}{2(2\pi)^6}\Bigg(-(\kappa_0\kappa_1^2+\kappa_0'\kappa_1'^2)\Big(-9+24\pi^2-24\pi^4+\eta(18-60\pi^2+20\pi^4)\Big)$$
$$-(\kappa_0+\kappa_0')\kappa_1\kappa_1'\Big(9+6\pi^2-20\pi^4+\eta(-18-30\pi^2+16\pi^4)\Big)\cos[2\alpha\pi]$$
$$+9\pi^3(\kappa_0-\kappa_0')\kappa_1\kappa_1'\big(-3+4\eta\big)\sin[2\alpha\pi]\Bigg) \tag{S.15}$$

Remarkably, if both flagella have the same magnitude of intrinsic curvature ($\kappa_0 = -\kappa_0'$) and beat at equal amplitude of dynamic mode ($\kappa_1 = -\kappa_1'$) with phase difference $2\pi\alpha$, then Eq. S.15 will be reduced to Eq. 15 and in the limit of $\alpha = 0$, it simplifies to Eq. 16.

Supplemental Movies and Figures

Video 1 Tracked trajectories plotted on top of experimental data for the first 800 msec.
Video 2 Trajectory of basal body obtained by a Gaussian fit showing a helical path.
Video 3 Video showing superposition of four eigenmodes on top of tracked flagellum.
Video 4 Swimming trajectory of BA obtained by RFT simulations.
Video 5 Simulations with simplified wave form with equal values of dynamic and static modes and frequency for both flagella and no phase difference (see Fig. 7A).
Video 6 Simulations with simplified wave form with equal values of dynamic and static modes and frequency but phase difference of $\pi/4$ (see Fig. 7B).
Video 7 Simulations with simplified wave form with equal values of dynamic and static mode but phase difference of $\pi/2$ (Fig. 7C).
Video 8 Simulations with simplified wave form with equal phase, κ_0, κ_1, but difference in frequency (see Fig. 8A).
Video 9 Simulations with simplified wave form with equal phase and κ_0, but different frequency and κ_1 (see Fig. 8B).
Video 10 Simulations with simplified wave form with equal phase, frequency and κ_0, but different κ_1 (see Fig. 8C).
Video 11 Same as Video 8 with $V_{angle} = 90°$ (see Fig. 8D).
Video 12 Same as Video 9 with $V_{angle} = 90°$ (see Fig. 8E).
Video 13 Same as Video 10 with $V_{angle} = 90°$ (see Fig. 8F).
Video 14 An intact wall-less *C. reinhardtii* cell attached via cell body to the substrate while its two flagella can beat freely.
Video 15 Another exemplary basal apparatus swimming in the vicinity of the substrate and is used for phase analysis in Fig. S5.
Video 16 A basal apparatus showing tumbling motion in 3D.
Video 17 A basal apparatus switching from 2D motion to 3D.
Video 18 A basal apparatus swimming in 3D with only one beating flagellum while the second one is not active.
Video 19 A basal apparatus swimming effectively in 2D with only one beating flagellum while the second one is not active.
Video 20 A basal apparatus swimming in a straight path with only one beating flagellum while the second one is not active.

Supplemental Figures S1-S5

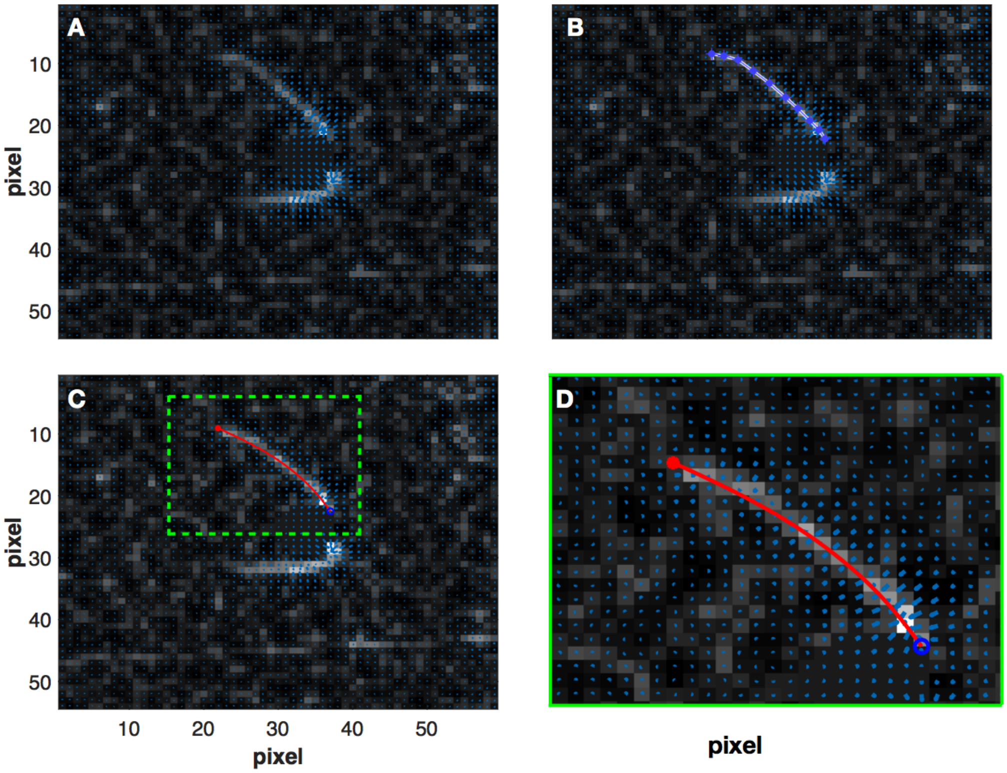

Fig.S 1 A) GVF (blue arrows) calculated around flagella apparatus. B) The initial selection of a polygon for the first frame which deforms according to the GVF calculated around the top flagellum. C) The final tracked shape of top flagella. D) A zoomed-in image showing GVF in higher magnification in the vicinity of top flagella.

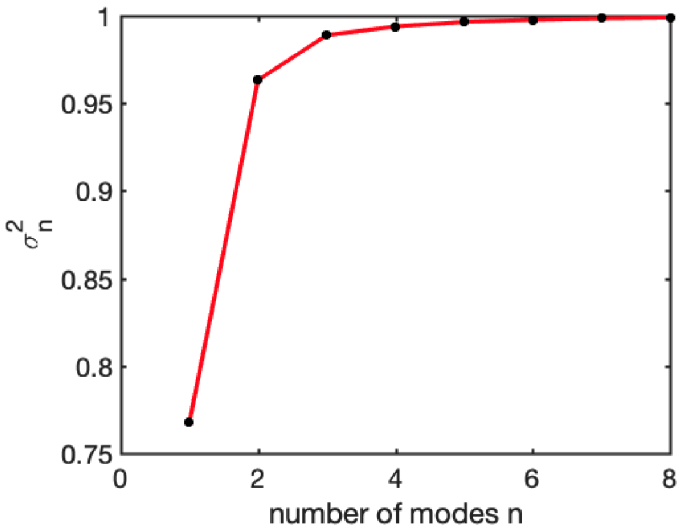

Fig.S 2 Fraction of the total variance σ_n^2 plotted versus the number of modes n. See section. 2.3 for the definition of σ_n^2. Note that already two modes capture 96% and four modes capture 99% of the total variance.

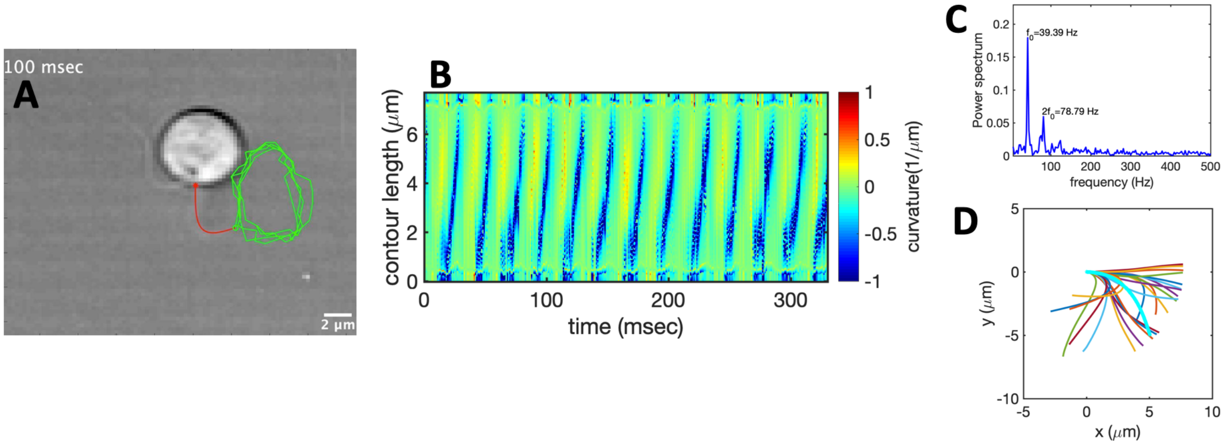

Fig.S 3 A) A sample snapshot of an intact *Chlamydomonas* cell (wall-less strain) which is attached via cell body to the substrate while its two flagella beat freely. One flagellum is tracked using GVF method. B) Curvature waves propagate from the basal proximal towards the distal tip. C) Power spectrum of curvature waves show a dominant peak at 40 Hz, and D) Mean shape of the tracked flagellum (filament in cyan color) has averaged curvature of 0.2 μm^{-1}, comparable to values in Fig. 1 for isolated basal apparatus. See also supplemental Video 14.

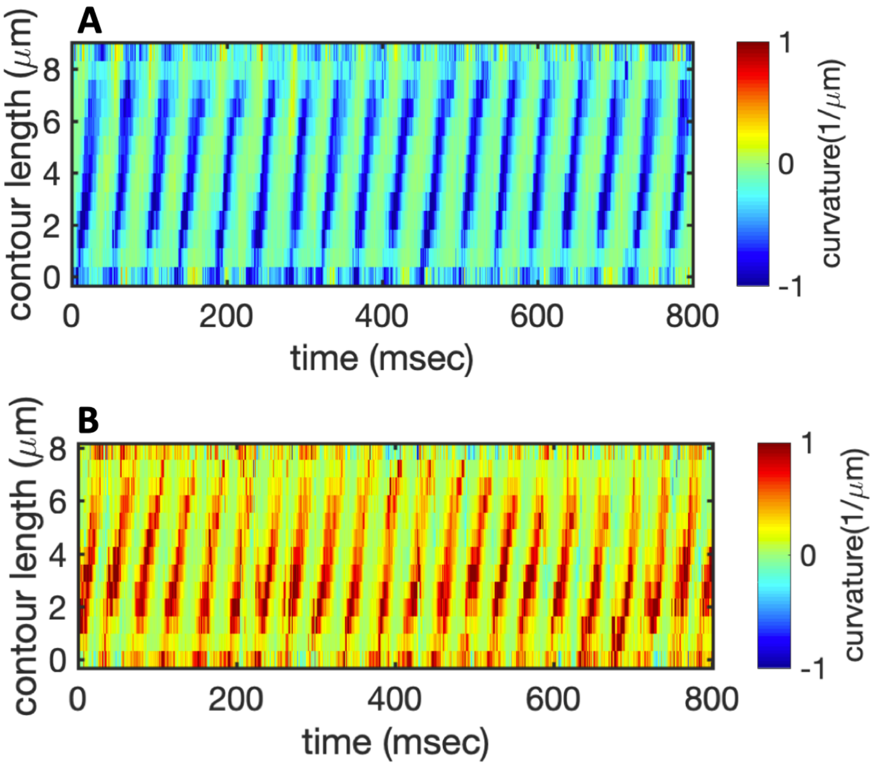

Fig.S 4 Curvature waves presented over longer time interval of 0 to 800 msec for A) flagella 1 and B) flagella 2 of the swimming basal apparatus in Fig. 1.

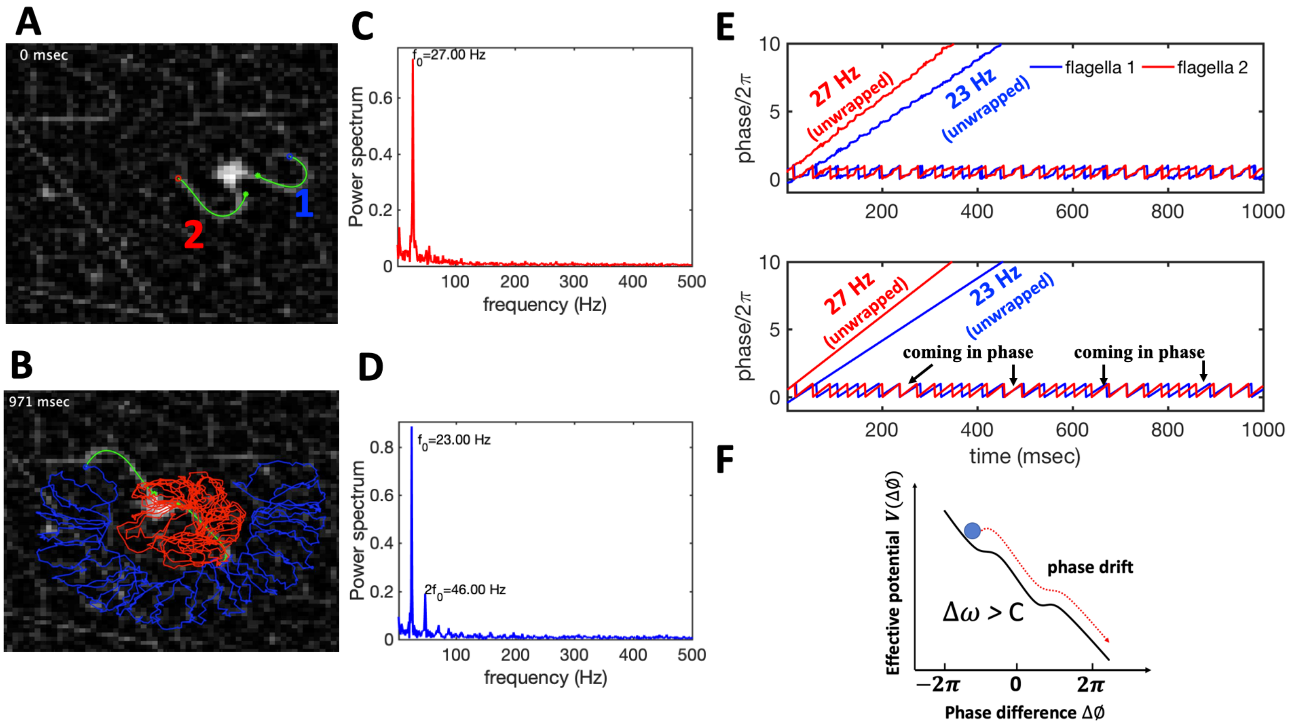

Fig.S 5 A-E) Phase analysis of another exemplary swimming basal apparatus showing similar dynamics as Fig. 4. Two flagella beat at different frequencies of 23 and 27 Hz. See also supplemental Video 15. F) For the case of large frequency mismatch which exceeds the synchronization strength C, two oscillators fail to synchronize and phase drifts occur, i.e. phase difference monotonically increases or decreases over time with slope $\Delta\omega$.

Table 1 Frequencies of N=10 basal apparatus considered in this work.

Basal Apparatus	1	2	3	4	5	6	7	8	9	10
f_1(Hz)	21	18.5	21.7	22.7	21.7	24.3	27.7	4.5	6	3.3
f_2(Hz)	25	20.4	25.6	26.3	26.3	30.6	20.8	2.9	8.8	5
Δf(Hz)	4	1.9	3.9	3.6	4.6	6.3	6.9	1.6	2.8	1.7
percentage of the mean	17.4%	9.8%	16.5%	14.7%	19.2%	22.9%	28.4%	43%	37.8%	41%
